# A succinate/SUCNR1-brush cell defense program in the tracheal epithelium

**DOI:** 10.1126/sciadv.adg8842

**Published:** 2023-08-02

**Authors:** Alexander Perniss, Brett Boonen, Sarah Tonack, Moritz Thiel, Krupali Poharkar, Mohamad Wessam Alnouri, Maryam Keshavarz, Tamara Papadakis, Silke Wiegand, Uwe Pfeil, Katrin Richter, Mike Althaus, Johannes Oberwinkler, Burkhard Schütz, Ulrich Boehm, Stefan Offermanns, Trese Leinders-Zufall, Frank Zufall, Wolfgang Kummer

**Affiliations:** ^1^ Institute of Anatomy and Cell Biology, German Center for Lung Research, Justus Liebig University Giessen; Giessen, Germany.; ^2^ Excellence Cluster The Cardio-Pulmonary Institute, Justus Liebig University Giessen, Giessen, Germany.; ^3^ Center for Integrative Physiology and Molecular Medicine, Saarland University, Homburg, Germany.; ^4^ Laboratory of Ion Channel Research, VIB Center for Brain and Disease, Department of Cellular and Molecular Medicine, Katholieke Universiteit Leuven, Leuven, Belgium.; ^5^ Department of Pharmacology, Max Planck Institute for Heart and Lung Research, Bad Nauheim, Germany.; ^6^ Laboratory of Experimental Surgery, Department of General and Thoracic Surgery, Justus-Liebig-University, Giessen, Germany.; ^7^ Physiology Group, Bonn-Rhein-Sieg University of Applied Sciences, Rheinbach, Germany.; ^8^ Institut für Physiologie und Pathophysiologie, Philipps-Universität Marburg, Marburg, Germany.; ^9^ Institute of Anatomy and Cell Biology, Philipps University Marburg, Marburg, Germany.; ^10^ Experimental Pharmacology, Center for Molecular Signaling (PZMS), Saarland University, Homburg, Germany.

## Abstract

Host-derived succinate accumulates in the airways during bacterial infection. Here, we show that luminal succinate activates murine tracheal brush (tuft) cells through a signaling cascade involving the succinate receptor 1 (SUCNR1), phospholipase Cβ2, and the cation channel transient receptor potential channel subfamily M member 5 (TRPM5). Stimulated brush cells then trigger a long-range Ca^2+^ wave spreading radially over the tracheal epithelium through a sequential signaling process. First, brush cells release acetylcholine, which excites nearby cells via muscarinic acetylcholine receptors. From there, the Ca^2+^ wave propagates through gap junction signaling, reaching also distant ciliated and secretory cells. These effector cells translate activation into enhanced ciliary activity and Cl^−^ secretion, which are synergistic in boosting mucociliary clearance, the major innate defense mechanism of the airways. Our data establish tracheal brush cells as a central hub in triggering a global epithelial defense program in response to a danger-associated metabolite.

## Teaser

Succinate triggers long-range Ca^2+ ^waves via tracheal brush cells to boost mucociliary clearance and chloride secretion.

## INTRODUCTION

Brush cells (also termed cholinergic chemosensory cells or tuft cells), a rare (~1%) cell type of the respiratory epithelium, hold a central position in surveilling the composition of the airway lining fluid and initiating defense reactions in response to pathogen-derived material. Their effector repertoire includes excitation of nearby sensory nerve fibers (*1*–*4*), promotion of type 2 immune responses (*5*), stimulation of antimicrobial peptide release *(**6**)*, and, recently, a link to mucociliary clearance, the major innate defense mechanism removing pathogens trapped by mucus from the airways, has been demonstrated (*7*, *8*). This process requires generation of a mechanical force to drive transport and adequate fluid secretion to hydrate and facilitate the flow of the viscous mucus. The required driving force is provided by ciliated cells, each of them being equipped with 100 to 200 motile cilia that all beat toward the larynx in a coordinated fashion, generating a directed flow of the lining fluid (*9*). To allow for effective transport, the cilia must move freely in a low viscous periciliary fluid, and mucus has to be adequately hydrated. This is achieved by active ion secretion, with water following passively. Chloride secretion by secretory cells holds a key position in the formation and regulation of the physicochemical properties of this watery component of the airway lining fluid (*10*–*12*). Both proper ciliary function and ion secretion are each indispensable for maintaining effective mucociliary clearance, as an isolated defect in only one of them results in overall insufficient clearing function with resulting pathologies. Recurrent respiratory infections are the hallmark feature of various forms of primary ciliary dyskinesia with abnormal motion of cilia (*13*) and of cystic fibrosis, one of the most common inherited diseases caused by mutations in the Cl^−^ and bicarbonate ion channel cystic fibrosis transmembrane conductance regulator (CFTR) (*14*).

In the mouse trachea, brush cell activation stimulates ciliary activity through paracrine release of acetylcholine (ACh), resulting in increased ciliary beat frequency (CBF) and accelerated particle transport speed (PTS) on the tracheal surface. Signal transduction in brush cells required phospholipase Cβ2 (PLCβ2) (*8*) and TRPM5 (transient receptor potential channel subfamily M member 5) (*7*, *8*), a monovalent cation channel originally identified in the downstream signaling cascade of type II taste cells in oropharyngeal gustation (*15*). As a trigger, we identified virulence-associated bacterial formyl peptides (*8*), and other bacterial products were also proposed (*7*). However, despite these advances, it remains unclear through which receptors brush cells detect danger-associated signals, how the spatially limited release of ACh from a rare epithelial cell type translates into a mass movement of fluid and particles on the mucosal surface, and whether this stimulation of ciliary activity is accompanied by adequate ion secretion, the second essential component of mucociliary clearance. We thus sought to identify a molecularly defined ligand-receptor pair that selectively activates brush cells in the mouse trachea, to visualize the spread of activity to both ciliated and secretory cells over the mucosal surface using high-resolution spatiotemporal Ca^2+^ imaging, and to determine the effects on ciliary activity and transepithelial ion transport.

## RESULTS

### *Sucnr1* is expressed by tracheal brush cells

**Fig. 1.**
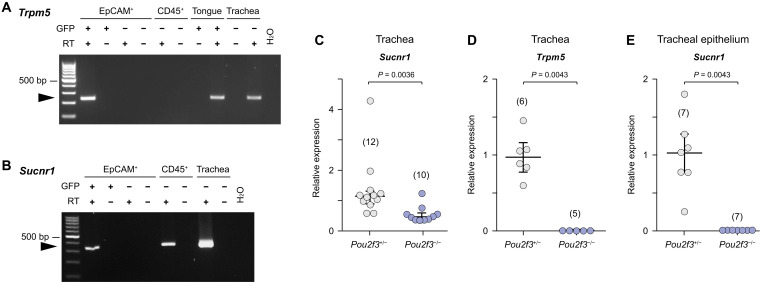
*Sucnr1* is exclusively expressed by brush cells in the tracheal epithelium. (**A** and **B**) Reverse transcription polymerase chain reaction (RT-PCR) of tracheal cells obtained by fluorescence-activated cell sorting (FACS) from *Trpm5*-GFP mice with primers specific for *Trpm5* (A) and *Sucnr1* (B), agarose gel electrophoresis. EpCAM, epithelial cell adhesion molecule (epithelial cell marker), CD45 (immune cell marker); RT ±, samples processed with and without reverse transcriptase; H_2_O, sample without cDNA; tongue and whole trachea served as positive controls. Representative of *n* = 2. (**C** to **E**) Relative expression of *Sucnr1* (C and E) and *Trpm5* (D) in whole tracheae (C and D) and mechanically abraded tracheal epithelium (E) of *Pou2f3*^+/−^ and *Pou2f3*^−/−^ mice assessed by real-time RT-PCR. Scatterplots show data, median and interquartile range (IQR), and the number of tracheae is given in parentheses, data from two independent experiments; Mann-Whitney test.

In search of a ligand-receptor pair activating tracheal brush cells, we focused upon succinate and short-chain fatty acids (SCFAs) as potential ligands, because their cognate receptors succinate receptor 1 (SUCNR1; synonym: GPR91) and free fatty acid receptor 2 (FFAR2; synonym: GPR43) are linked to TRPM5 in intestinal (*16*, *17*) and biliary tuft cells (*18*). Succinate accumulates in substantial amounts in the bronchoalveolar lavage fluid during bacterial infection (*19*). Therefore, we first assessed the expression of the succinate receptor SUCNR1. We performed reverse transcription polymerase chain reaction (RT-PCR) experiments of isolated and sorted tracheal cells obtained from *Trpm5*-eGFP mice in which all TRPM5^+^ brush cells are genetically labeled by green fluorescent protein (GFP) (*20*). These analyses revealed that *Sucnr1* is expressed by both immune cells (CD45^+^ and GFP^−^) and brush cells [epithelial cell adhesion molecule^+^ (EpCAM^+^) and GFP^+^] but not by other tracheal epithelial cells (EpCAM^+^ GFP^−^) (Fig. 1, A and B). Brush cell identity was confirmed by the presence of *Trpm5*-mRNA exclusively in GFP^+^ cells (Fig. 1A and fig. S1, A to C). We confirmed these results by analyzing published single-cell RNA sequencing data (*21*), showing that *Sucnr1* was present only in brush cells but not in other airway epithelial cell types (fig. S1, D to G). Furthermore, *Sucnr1* was reduced by ~60% in tracheae from brush cell–deficient mice (*Pou2f3^−/−^*), which lack the TRPM5^+^ brush cells (Fig. 1, C and D) (*22*), and was not detected in tracheal epithelial cells of *Pou2f3^−/−^* mice (Fig. 1E), indicating that the brush cells are the sole source of *Sucnr1* expression in the tracheal epithelium.

The SCFA receptor *Ffar2* was weakly expressed in basal, club, and neuroendocrine cells but not detected in brush cells, and expression of *Ffar3* was not observed at all (fig. S1, H and I). Thus, *Sucnr1* is present in tracheal TRPM5^+^ brush cells, where it could function as a sensor of its cognate ligand succinate.

### Tracheal brush cells are succinate sensors

**Fig. 2.**
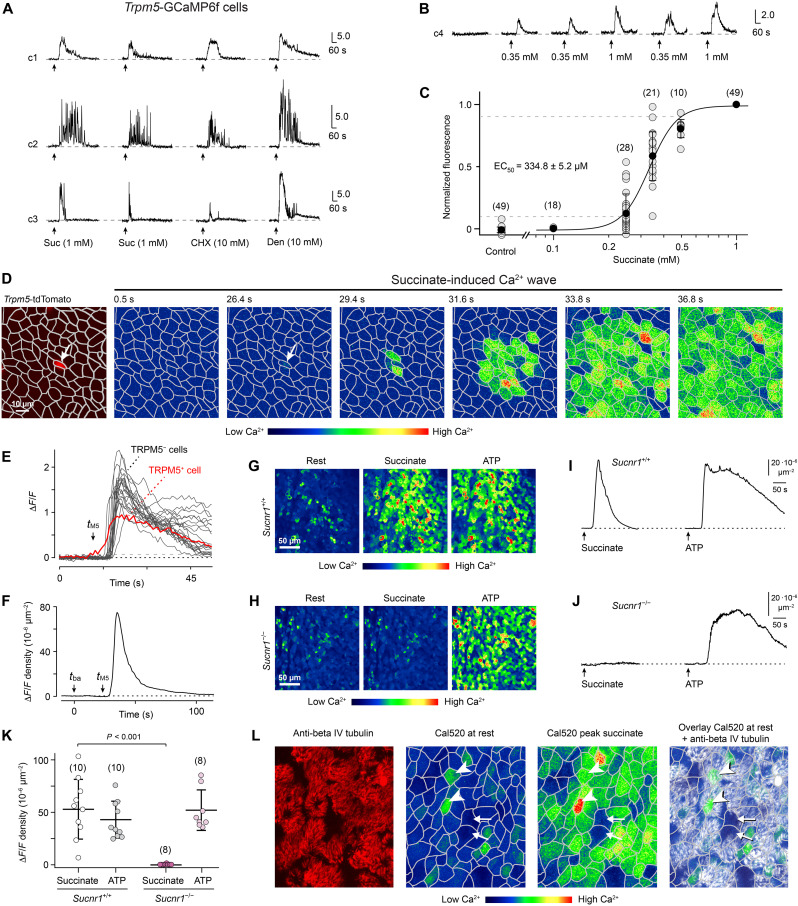
Succinate/SUCNR1 sensing by brush cells triggers long-range Ca^2+^ waves across the tracheal epithelium. (**A**) Ca^2+^ transients in individual *Trpm5*-GCaMP6f brush cells (c1 to c3) to succinate (Suc), cycloheximide (CHX), and denatonium (Den). Seventy-two of 86 cells responded to succinate, 73 of 86 cells to cycloheximide, and 83 of 86 cells to denatonium. (**B** and **C**) Concentration dependence of succinate-evoked Ca^2+^ responses in TRPM5^+^ cells and dose-response curve of their peak values. (**D**) Succinate-evoked intercellular Ca^2+^ wave shown as a two-dimensional (2D) Ca^2+^ activity pattern at different time points. Arrow, TRPM5^+^ cell. (**E**) The Ca^2+^ response of a TRPM5^+^ cell precedes those of neighboring cells after succinate stimulation. Dashed lines: black, baseline (Fb); gray, mean Fb + 2SD. (**F**) Δ*F*/*F* density of succinate-evoked Ca^2+^ wave recovered after ~80 s (*t*_ba_, start of bath application; *t*_M5_, start of TRPM5^+^ cell response). (**G** to **K**) Examples of epithelial peak Ca^2+^ images to succinate or adenosine triphosphate (ATP) [(G) and (H)], their time courses [(I) and (J)], and group data (K) summarizing Δ*F*/*F* density peak analyses (mean ± SD) in *Sucnr1*^+/+^ versus *Sucnr1*^−/−^ mice (*n* = 5 per genotype). Analysis of variance (ANOVA), posthoc: Tukey. (**L**) Ciliated cells (anti-beta IV tubulin, red, white in overlay) mostly have higher Ca^2+^ levels (arrowheads) than nonciliated cells (arrows); both respond to succinate with a Ca^2+^ increase. Gray lines, cell borders [(D) and (L)]. Succinate, 1 mM; ATP, 20 μM. Numbers in parentheses indicate independent experiments.

We used Ca^2+^ imaging in identified TRPM5^+^ brush cells to test whether these cells are functional succinate sensors. For this approach, we used an acutely isolated (ex vivo) tracheal whole-mount preparation in which the fast genetically encoded Ca^2+^ sensor GCaMP6f is expressed selectively in TRPM5^+^ cells (*Trpm5*-IRES-Cre/ROSA26-GCaMP6f mice) and in which temporally and spatially resolved Ca^2+^ signals can be recorded through confocal imaging in an intact epithelium (*8*). Exposure to succinate (1 mM) produced repeatable, transient Ca^2+^ elevations in nearly all of the TRPM5^+^ cells (72/86, 83.4%; *n* = 6 preparations) (Fig. 2A). Most of these cells responded also to the fungicide cycloheximide (10 mM), which is produced by *Streptomyces griseus*, and to the bitter substance denatonium (10 mM) (Fig. 2A).

To determine the sensitivity of succinate responses in TRPM5^+^ cells, we performed concentration-response measurements (Fig. 2, B and C). These results revealed a steep overall concentration-response curve with a 10 to 90% operating range spanning less than one order of magnitude, a Hill coefficient of 5.8 ± 0.5, and a median effective concentration (EC_50_) of 335 ± 5 μM. Together, these results identified the tracheal brush cells as sensors that recognize the metabolite succinate and respond with intracellular Ca^2+^ elevations.

### Brush cell stimulation by succinate/SUCNR1 triggers long-range Ca^2+^ waves

**Fig. 3.**
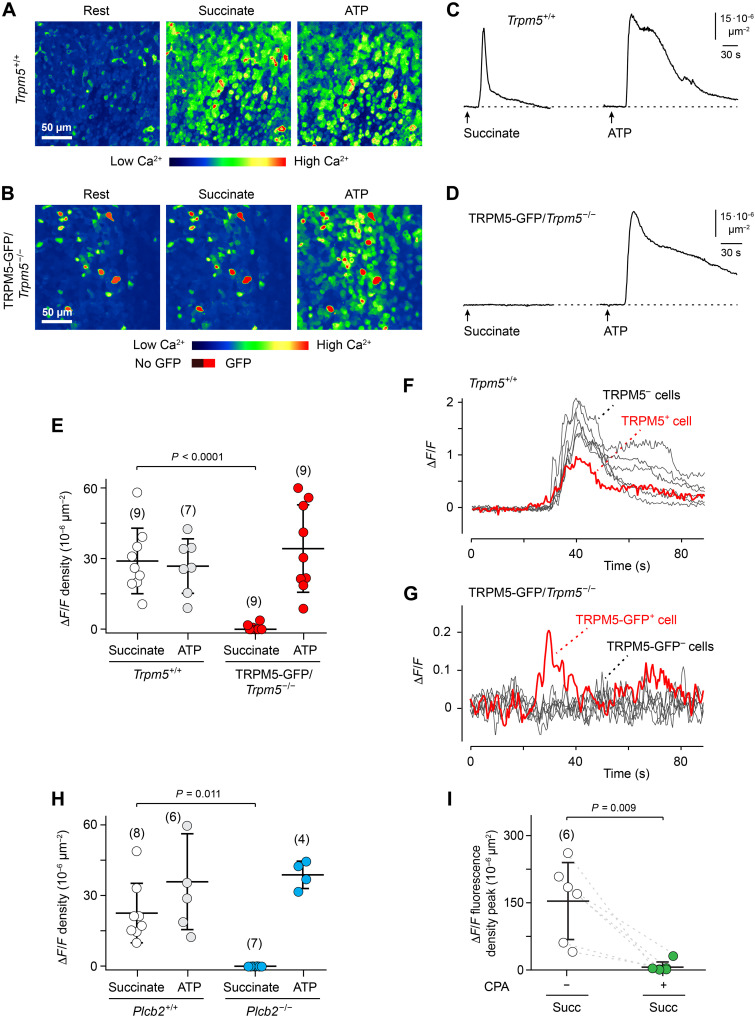
Genetic deletion of *Trpm5* or *Plcb2* or Ca^2+^ store depletion eliminate succinate-evoked Ca^2+^ waves. (**A** to **E**) Images of mean peak Ca^2+^ waves, time courses of the Ca^2+^ waves, and summary of Δ*F*/*F* density peak analyses (mean ± SD) at rest and during succinate- or adenosine triphosphate (ATP)–evoked responses in *Trpm5*^+/+^ (seven mice) versus TRPM5-GFP/*Trpm5*^−/−^ tracheae (six mice). Analysis of variance (ANOVA), posthoc: Tukey. (**F** and **G**) Time courses of succinate-evoked Ca^2+^ responses in a brush cell and its adjacent epithelial cells in tracheae from a *Trpm5^+/+^* (F) and a TRPM5-GFP/*Trpm5*^−/−^ trachea (G). (**H**) Δ*F*/*F* density peak measurements (mean ± SD) of succinate-evoked Ca^2+^ waves in *Trpm5*-tdTomato;*Plcb2*^+/+^ versus *Trpm5*-tdTomato;*Plcb2*^−/−^ tracheae. Original recordings for these experiments are depicted in fig. S3. ANOVA, posthoc: Tukey. (**I**) Δ*F*/*F* density peak measurements of succinate- and ATP-evoked Ca^2+^ responses before and during treatment with cyclopiazonic acid (CPA) (30 μM) (four mice). Succinate, 1 mM; ATP, 20 μM. Numbers in parentheses indicate independent experiments.

**Fig. 4.**
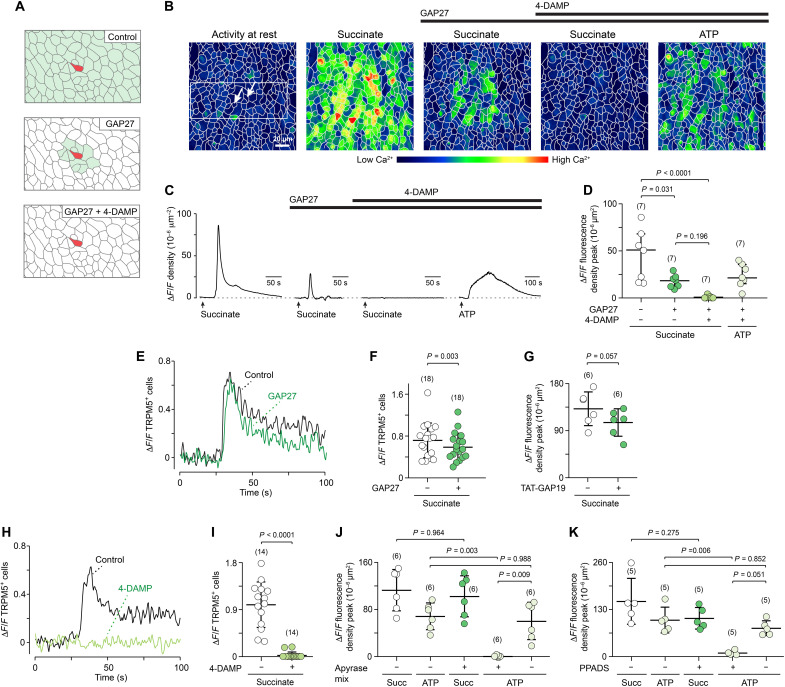
Propagation of succinate-evoked Ca^2+^ waves requires sequential cholinergic transmission and gap junction communication. (**A**) A model of succinate-evoked Ca^2+^ wave propagation in the trachea that predicts sequential activity of cholinergic transmission and long-range gap junction communication (see explanation in text). TRPM5^+^ trigger cell, red; succinate-evoked Ca^2+^ activity in epithelial cells, light green. (**B** and **C**) Example of the spatiotemporal properties of succinate-evoked Ca^2+^ waves and the effects of GAP27 (130 μM) or a combination of GAP27 and 4-DAMP (1 μM). Borders of cells, light gray. Time course analyses of individual cells within white box (B) are depicted in fig. S6 (A and B). Arrows, TRPM5^+^ cells. (**D**) Group data of experiments as shown in (C). Friedman analysis of variance (ANOVA), Dunn’s. Data, median ± interquartile range (IQR). (**E** to **I**) Original examples and group data showing succinate-evoked Ca^2+^ responses in TRPM5^+^ cells before and after treatment with GAP27 (130 μM), TAT-GAP19 (100 μM), or 4-DAMP (1 μM). Paired *t* test. Data, mean ± SD. (**J** and **K**) Δ*F*/*F* density peak values (mean ± SD) of succinate-evoked Ca^2+^ waves after treatment with either apyrase mix (apyrase VI and VII, 5 U/ml each) or pyridoxalphosphate-6-azophenyl-2′,4′-disulfonic acid (PPADS) (100 μM). Succinate, 1 mM; adenosine triphosphate (ATP), 20 μM. ANOVA, posthoc: Tukey. Numbers in parentheses indicate independent experiments.

We hypothesized that a localized activation of TRPM5^+^ cells by succinate could spread within the tracheal epithelium to link this activation with critical airway host defense mechanisms. To visualize a potential spread of activity with high spatial and temporal resolution in the intact epithelium, we developed a Ca^2+^ imaging procedure to record succinate-evoked intracellular Ca^2+^ activity in virtually every cell of the tracheal epithelium (Fig. 2, D to L). For this approach, we used *Trpm5*-IRES-Cre/ROSA26-tdTomato mice (referred to as *Trpm5*-tdTomato) for the whole-mount preparations, which enabled the identification of TRPM5^+^ cells by red fluorescence. In addition, we loaded the tissue with the fluorogenic Ca^2+^ indicator Cal-520 AM, which exhibits improved intracellular retention and signal-to-noise ratio compared to other existing green Ca^2+^ indicators (*23*, *24*). Combined with confocal microscopy, this method enabled stable visualization of repeated agonist-evoked Ca^2+^ activity across the entire field of view (FOV) for up to 2 to 3 hours (Figs. 2, 3, and 4).

Succinate exposure produced long-range, intercellular Ca^2+^ waves that originated in the TRPM5^+^ brush cells and subsequently spread radially across the tracheal epithelium. Examples of such waves are depicted as two-dimensional (2D) spatiotemporal Ca^2+^ activity patterns (Fig. 2D), as 3D activity patterns (fig. S2, A and B), and as original video recordings (movie S1). Such responses could be repeated several times in a given preparation without run-down (fig. S2C). Succinate-evoked Ca^2+^ elevations were initiated in the TRPM5^+^ cells and appeared later in the surrounding TRPM5^−^ cells (Fig. 2E). To capture the spatiotemporal properties of these waves quantitatively, we analyzed Δ*F*/*F* values per standard FOV (Δ*F*/*F* density; with a FOV of 15,134 μm^2^) over time (Fig. 2F and fig. S2, A and B). Additional experiments using larger FOVs with up to 544,792 μm^2^ revealed that the waves could cover epithelial areas of >200,000 μm^2^ (*n* = 7 mice) and traveled with speeds ranging between 2.1 and 15.8 μm/s (*n* = 19 waves in 11 mice), thus classifying the tracheal succinate-evoked Ca^2+^ waves as fast waves (*25*).

We tested whether the succinate-evoked Ca^2+^ waves required SUCNR1 by using mice with a constitutive knockout of *Sucnr1* (fig. S2, D and E). These experiments used adenosine triphosphate (ATP)–evoked epithelial Ca^2+^ elevations (20 μM ATP) as positive controls (see also movie S2). In *Sucnr1*^−/−^ epithelium, succinate-evoked Ca^2+^ waves were absent, whereas ATP-evoked Ca^2+^ responses remained unaffected. Responses to either stimulus were normal in *Sucnr1*^+/+^ epithelium (Fig. 2, G to K). Thus, SUCNR1 is required for the generation of succinate-evoked long-range Ca^2+^ waves, while the absence of SUCNR1 does not alter ATP-evoked responses. These results also provided further evidence that the succinate-evoked Ca^2+^ waves originated from the TRMP5^+^ brush cells, which selectively express *Sucnr1*.

We performed post hoc immunohistochemistry of the tracheal epithelium after Ca^2+^ imaging using antibodies that recognize the ciliated cell population (anti-beta IV tubulin) and then superimposed anatomical images onto the Ca^2+^ activity patterns (Fig. 2L). These experiments, together with other data shown below, revealed that succinate exposure eventually caused the activation of almost every cell in the FOV including the ciliated and nonciliated cells, the latter of which are composed of >90% of secretory cells. Together, these results show that localized activation of the tracheal brush cells by succinate spreads to virtually every other cell in the tracheal epithelium to evoke additional responses by those cells and thus produces massive feed-forward activation.

### TRPM5, PLCβ2, and Ca^2+^ stores are indispensable for intercellular tracheal Ca^2+^ waves

We hypothesized that the Ca^2+^-activated monovalent cation channel TRPM5 could be a key ion channel in the initiation of the succinate-evoked Ca^2+^ waves by the tracheal brush cells. To test this, we used transgenic mice expressing GFP under the control of the *Trpm5* promoter in a *Trpm5* knockout background (TRPM5-GFP/*Trpm5*^−/−^) (*26*–*28*), in which brush cells are genetically labeled by GFP irrespective of TRPM5 expression, allowing us to identify TRPM5-deficient brush cells. We then analyzed 2D spatiotemporal Ca^2+^ activity patterns (Fig. 3, A and B), Δ*F*/*F* density (Fig. 3, C to E), and the Ca^2+^ time courses of individual brush cells and surrounding epithelial cells (Fig. 3, F and G) in *Trpm5*^+/+^ versus *Trpm5*^−/−^ tracheae. Succinate-evoked Ca^2+^ waves were abolished in *Trpm5*^−/−^ tracheae (Fig. 3, B, D, and E), whereas the brush cells could still produce succinate-evoked Ca^2+^ transients under these conditions (Fig. 3, F and G). By contrast, ATP-evoked Ca^2+^ responses remained unaltered in *Trpm5*^−/−^ tracheae (Fig. 3, B, D, and E). Thus, we conclude that TRPM5-mediated depolarization of the tracheal brush cells provides a critical step in the initiation of succinate-evoked Ca^2+^ waves.

We next tested a role for the enzyme PLCβ2 (gene name *Plcb2*) in Ca^2+^ wave generation, which is likely to function in the brush cell signaling cascade downstream of SUCNR1 and upstream of TRPM5 (*29*, *30*). We crossed the *Trpm5*-tdTomato mice with mice harboring a constitutive knockout of *Plcb2* (*8*) and analyzed the spatiotemporal properties of succinate-evoked Ca^2+^ waves in *Plcb2*^+/+^ versus *Plcb2*^−/−^ tracheae. These experiments revealed that succinate-evoked Ca^2+^ waves were abolished in *Plcb2*^−/−^ tracheae, whereas ATP-evoked Ca^2+^ responses remained unaltered (Fig. 3H and fig. S3, A to D).

We also tested the impact of intracellular Ca^2+^ stores on succinate-evoked Ca^2+^ waves. Removal of the endoplasmic reticulum Ca^2+^ release component by inhibiting sarcoplasmic/endoplasmic reticulum calcium adenosine triphosphatase (ATPase) (SERCA) with cyclopiazonic acid (CPA; 30 μM) (*31*) abolished succinate-evoked Ca^2+^ waves (Fig. 3I). Thus, Ca^2+^ wave generation by succinate requires store-dependent Ca^2+^ release. Together, these results identified SUCNR1, PLCβ2, store-dependent Ca^2+^ release, and TRPM5 as essential for the generation of succinate-evoked Ca^2+^ waves in the trachea.

### Ca^2+^ waves propagate by sequential mechanisms in the trachea

How do the succinate-evoked Ca^2+^ waves propagate from the brush cells across the tracheal epithelium? We previously showed that brush cells stimulated by bacterial peptides enhance ciliary activity through the release of ACh (*8*). In view of the long distances bridged by the succinate-induced Ca^2+^ waves, we questioned that this will be solely caused by diffusion of ACh, and we assumed an additional communication route. Major pathways of Ca^2+^ signal propagation among airway epithelial cells are direct gap junction communication (*6*, *32*) and hemichannel-mediated release of 5′-nucleotide-triphosphates (ATP and uridine triphosphate) acting upon purinergic P2 receptors (*33*, *34*). Accordingly, in silico analyses of single-cell RNA sequencing data [dataset GSE102580; (*35*)] revealed abundant expression of connexins (Cx26, Cx31, Cx32, Cx40, Cx43, and Cx50) and P2 receptors (P2X2, P2X4, P2X7, P2Y1, and P2Y2) in secretory cells and a smaller spectrum (mainly Cx50, P2X4, and P2X7) in ciliated cells. Brush cells expressed connexins only very weakly, and P2X4, also previously detected in tracheal brush cells (*36*), dominated among purinergic receptors (figs. S4 and S5).

We, thus, proposed a sequential model in which wave propagation depends on two principal, consecutive events: (i) paracrine ACh secretion from the TRPM5^+^ cells followed by activation of neighboring TRPM5^−^ cells; and (ii) gap junction/hemichannel communication between the ACh-activated cells and distant regions of the epithelium (Fig. 4A). This model predicts that blockade of gap junctions/hemichannels should eliminate a large portion of the succinate-evoked Ca^2+^ wave but should leave intact a much smaller paracrine component of Ca^2+^ responses in cells close to the TRPM5^+^ brush cells which, in turn, should be blocked by ACh receptor (AChR) antagonists (Fig. 4A).

We tested these predictions by imaging consecutive succinate-evoked Ca^2+^ waves under (i) control conditions; (ii) in the presence of GAP27 (SRPTEKTIFII, 130 μM), a Cx43 mimetic peptide that also binds to Cx32 and Cx40 and potently inhibits gap junction-mediated Ca^2+^ wave propagation after mechanical stimulation (*37*, *38*); and (iii) in the presence of GAP27 and 1,1-dimethyl-4-diphenylacetoxypiperidinium iodide (4-DAMP; 1 μM), a muscarinic AChR antagonist (*39*). GAP27 treatment inhibited the succinate-evoked Ca^2+^ waves to a large extent but left intact residual Ca^2+^ responses in a smaller subset of both ciliated and nonciliated cells located close to the TRPM5^+^ trigger cells (Fig. 4, B to D, and fig. S6, A to C). In this example, succinate initially activated 318 of 331 cells (99.1%) under control conditions which declined to 35 cells (10.9%) after GAP27 treatment. GAP27 treatment did not affect the original succinate-evoked Ca^2+^ signal within the brush cells (Fig. 4, E and F, and fig. S6B). The peptide TAT-GAP19 (YGRKKRRQRRRKQIEIKKFK, 100 μM), which inhibits Cx43 hemichannels without blocking gap junctions or Cx40/pannexin1 hemichannels (*40*), did not inhibit succinate-evoked Ca^2+^ waves (Fig. 4G).

Consistent with the model of Fig. 4A, additional treatment with 4-DAMP abolished the residual succinate-evoked Ca^2+^ waves (Fig. 4, B to D). However, 4-DAMP treatment also caused a strong reduction or even a complete loss of the original succinate-evoked Ca^2+^ signal within the TRPM5^+^ brush cells (Fig. 4, G and H, and fig. S6B). Closely similar results were obtained with the muscarinic AChR inhibitor atropine (10 μM) or a mixture of atropine and the nicotinic AChR inhibitor mecamylamine (fig. S6, E to H). These diminished responses to succinate were not attributable to off-target inhibitory effects of atropine or 4-DAMP on SUCNR1, as shown in a SUCNR1 overexpressing cell line (fig. S7). One likely explanation of these results is that ACh release by the TRMP5^+^ brush cells does not only lead to paracrine effects through muscarinic AChR activation but also to autocrine (i.e., positive feedback) effects onto the trigger cells themselves. In support of this assumption, ACh evoked an increase in intracellular [Ca^2+^] in *Trpm5*-GCamP6f brush cells (fig. S6D). Positive cholinergic feedback via muscarinic receptors has also previously been shown for isolated tracheal brush cells. They release ACh upon activation with the broad stimulant denatonium, react themselves to ACh with an increase in intracellular [Ca^2+^], and show largely diminished Ca^2+^ responses to denatonium in the presence of atropine (*7*).

There was no evidence for ATP release driving the succinate-evoked Ca^2+^ waves by acting upon purinergic P2 receptors, because neither treatment with apyrase, an enzyme that degrades the purines ATP and adenosine diphosphate (ADP) (*41*), nor broad P2 receptor inhibition by pyridoxalphosphate-6-azophenyl-2′,4′-disulfonic acid (PPADS; 100 μM) had an impact on succinate-evoked Ca^2+^ waves, whereas ATP-evoked Ca^2+^ responses were inhibited (Fig. 4, J and K, and fig. S6I). Still, this does not exclude the possibility that ATP might be released under these conditions and serves redundant function.

These results provide strong support for a combined paracrine/autocrine and gap junction model in the propagation of succinate-evoked Ca^2+^ waves. In this scenario, a first step depends on ACh secretion by the TRPM5^+^ brush cells followed by ACh detection through muscarinic AChRs, and a subsequent step requires gap junction communication between the major epithelial cell types.

### Succinate boosts ciliary activity via brush cells and sequential cholinergic and gap junction signaling

**Fig. 5.**
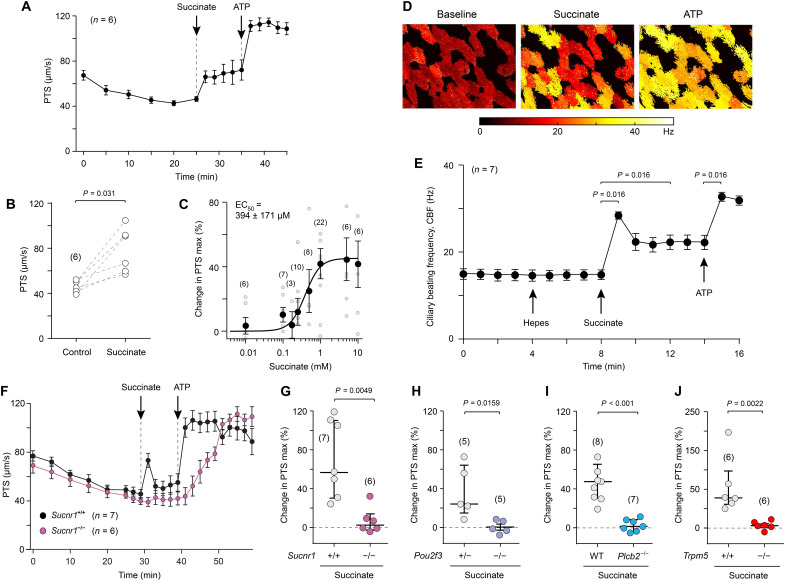
Succinate drives ciliary activity through SUCNR1 signaling in brush cells. (**A**) Particle transport speed (PTS) at the murine tracheal surface over time in response to succinate (1 mM) and adenosine triphosphate (ATP) (100 μM; viability control). (**B**) PTS before (control) and 2 min after succinate administration in experiments depicted in (A), Kruskal-Wallis-test. (**C**) Maximum changes in PTS, concentration-response curve to increasing concentrations of succinate. (**D** and **E**) Ciliary beat frequency (CBF) before and after application of succinate and adenosine triphosphate (ATP) (viability control). (D) Representative images of the tracheal surface, false-color coding of CBF. (E) CBF over time, Hepes: vehicle control. (**F**) PTS recorded over time from tracheae of *Sucnr1^−/−^* mice and their wild-type littermates (*Sucnr1^+/+^*) before and after succinate and ATP stimulation. (**G** to **J**) Maximum changes in PTS evoked by succinate in tracheae with genetic deletion of *Sucnr1* (G), of the transcription factor *Pou2f3*, resulting in lack of brush cells (H), and of the signal transduction elements *Plc*β*2* (I) and *Trpm5* (J); controls were heterozygous (H) and homozygous wild-type littermates [(G) and (J)] and wild-type C57BL6/J mice (I). Mann-Whitney test. (A) to (F) Data, mean ± SEM. (G) to (J) Data, median ± interquartile range (IQR). Succinate, 1 mM; ATP, 100 μM. Numbers in parentheses indicate independent experiments (= tracheae).

Since the succinate-induced epithelial Ca^2+^ wave reached the ciliated cells, we hypothesized that this signal may promote ciliary activity, the major driver of mucosal clearance. Testing this hypothesis in explanted tracheae, we observed that succinate (1 mM) triggered a rapid acceleration of PTS on the mucosal surface from 48 to 65 μm/s within the first 2 min after application, which was further accelerated by ATP (Fig. 5, A and B). The concentration-response curve of this effect was steep, with a 10 to 90% operating range between 0.1 and 1 mM and an EC_50_ of 394 ± 171 μM (Fig. 5C), consistent with the Ca^2+^ imaging experiments (Fig. 2, B and C). Increased ciliary activity was also reflected by an acute rise in CBF upon succinate (1 mM) administration in the explanted trachea preparation (Fig. 5, D and E). In contrast, neither of the SCFAs acetate, propionate, and butyrate induced an increase in PTS (fig. S8, A to C), consistent with the lack of expression of their cognate receptors FFAR2 and FFAR3 in both brush and ciliated cells (fig. S1, H and I).

**Fig. 6.**
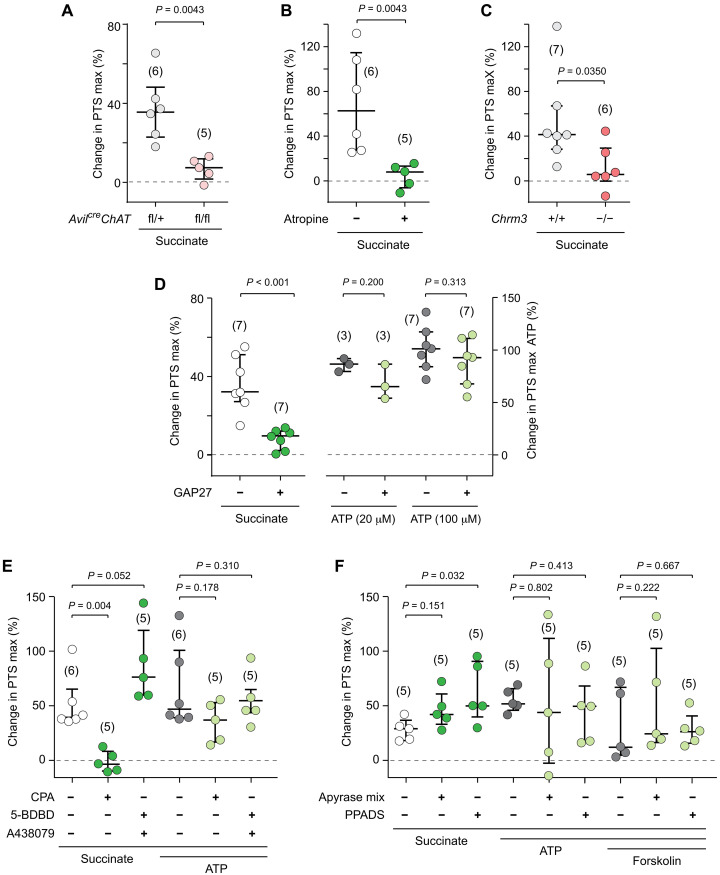
Succinate drives ciliary activity through sequential cholinergic and gap junction signaling. (**A** to **F**) Maximum changes in particle transport speed (PTS) evoked by succinate in tracheae with brush cell–specific deletion of the acetylcholine (ACh)–synthesizing enzyme *Chat* (*Avil^Cre^Chat^fl/fl^*) (A) or global deletion of the M3 muscarinic receptor (*Chrm3^−/−^*) (B), under general muscarinic receptor blockade with atropine (10 μM) (C), gap junction inhibition by GAP27 (130 μM, given 45 min before succinate) (D), SERCA inhibition by cyclopiazonic acid (CPA) (30 μM) (E), P2X4 and P2X7 inhibition by 5-BDBD (10 μM) and A438079 (20 μM) (E), general P2 receptor inhibition by pyridoxalphosphate-6-azophenyl-2′,4′-disulfonic acid (PPADS) (100 μM) (F), and apyrase treatment (apyrase VI and VII, 5 U/ml each) (F). Control conditions were heterozygous littermates (*Avil^Cre^Chat^fl/+^*) (A), homozygous wild-type animals from the same strain (*Chrm3^+/+^*) (C), and respective vehicle treatment [(B) and (D) to (F)]. Mann-Whitney test. Data, median ± interquartile range (IQR). Succinate, 1 mM; adenosine triphosphate (ATP), 100 μM, if not indicated otherwise (D). Numbers in parentheses indicate independent experiments (= tracheae).

The Ca^2+^ imaging experiments had demonstrated activation of TRPM5^+^ brush cells by succinate through its cognate receptor SUCNR1 and downstream signaling involving PLCβ2 and TRPM5, resulting in a Ca^2+^ wave in the epithelial layer (Figs. 2 and 3). We reasoned that this sequence of events also underlies the acceleration of PTS triggered by succinate and analyzed tracheae from different knockout mice, including mice lacking TRPM5^+^ brush cells due to deletion of the transcription factor *Pou2f3* (*22*). In support of our hypothesis, SUCNR1, brush cells, PLCβ2, and TRPM5 were all required for succinate-evoked acceleration of PTS (Fig. 5, F to J). We recently showed that activated brush cells can stimulate ciliary activity through paracrine release of ACh acting upon the muscarinic AChR M3 (M3R, gene name *Chrm3*) (*8*), the major AChR linked to cilia-driven particle transport in the mouse trachea (*42*). Likewise, succinate had nearly no effect on PTS when ACh synthesis was selectively abrogated in brush cells by cell type–specific deletion of its synthesizing enzyme, choline acetyltransferase (*Chat*), using the *advillin* promotor [*Avil^Cre^Chat^fl/fl^* (*7*)] (Fig. 6A), when the general muscarinic receptor antagonist atropine was applied (Fig. 6B) and when M3R was constitutively deleted (*Chrm3^−/−^*) (Fig. 6C). These data demonstrated that boosting ciliary activity by succinate required brush cell–derived ACh and M3R activation.

Our Ca^2+^ imaging experiments had shown that paracrine cholinergic signaling initiates the generation of a long-range Ca^2+^ wave, most likely propagated through gap junctions, and we hypothesized that this drives ciliary stimulation. Accordingly, the SERCA inhibitor CPA (30 μM) and the gap junction blocker GAP27, which suppressed Ca^2+^ wave propagation, largely suppressed the stimulatory effect of succinate on PTS, whereas that of ATP, which activates ciliated cells independent of brush cells, was not significantly reduced (Fig. 6, D and E). In contrast, there was no evidence for involvement of succinate-induced release of ATP acting upon purinergic P2 receptors, because neither apyrase treatment, nor blockade of the two highest expressed P2 receptors in ciliated cells, P2X4 and P2X7 (fig. S5), by 5-(3-bromophenyl)-1,3-dihydro-2H-benzofuro [3,2-e]-1,4-diazepin-2-one (5-BDBD; 10 μM) and A439079 (20 μM), nor broad P2 receptor inhibition by PPADS (100 μM) had an impact on succinate-induced increase in PTS (Fig. 6, E and F). Notably, the effect of exogenously applied ATP was also not significantly reduced, indicating that ATP metabolites such as adenosine also drive ciliary activity, as reported earlier (*43*–*44*). Collectively, these experiments revealed that succinate activates cilia-driven transport on the mucosal surface through a sequence of events that includes activation of brush cells through SUCNR1, downstream signaling involving PLCβ2 and TRPM5 to evoke paracrine ACh release, which then excites M3R-expressing neighboring cells from which a Ca^2+^-wave spreads in the epithelial layer through gap junction communication.

### The SUCNR1–brush cell pathway drives epithelial Cl^−^ secretion

The long-range Ca^2+^ wave evoked by succinate encompassed both ciliated and nonciliated cells. We, therefore, hypothesized that stimulation of ciliary activity will be paralleled by secretory events, in particular luminal ion secretion, which is the driving force of production of the watery periciliary fluid that allows for effective ciliary stroke. We measured transepithelial short-circuit current (*I*_SC_) as an indicator of ion transport in freshly isolated, opened tracheae mounted in bicarbonate buffered solution in an Ussing chamber. We first characterized the preparation using established activators of *I*_SC_ (100 μM nicotine, 100 μM ACh, and 10 μM forskolin) and measured suppression of currents induced by forskolin and reduction of those evoked by ACh by the nonselective Cl^−^ channel inhibitor 5-nitro-2-[3-phenylpropylamino]benzoic acid (NPPB; 100 μM). d-Mannitol (1 mM) was used as a control for osmolarity effects (fig. S9). The results of these experiments, including a short increase in *I*_SC_ induced by NPPB alone (fig. S9C), fully matched previous findings (*45*, *46*) and validated the functionality of the preparation.

**Fig. 7.**
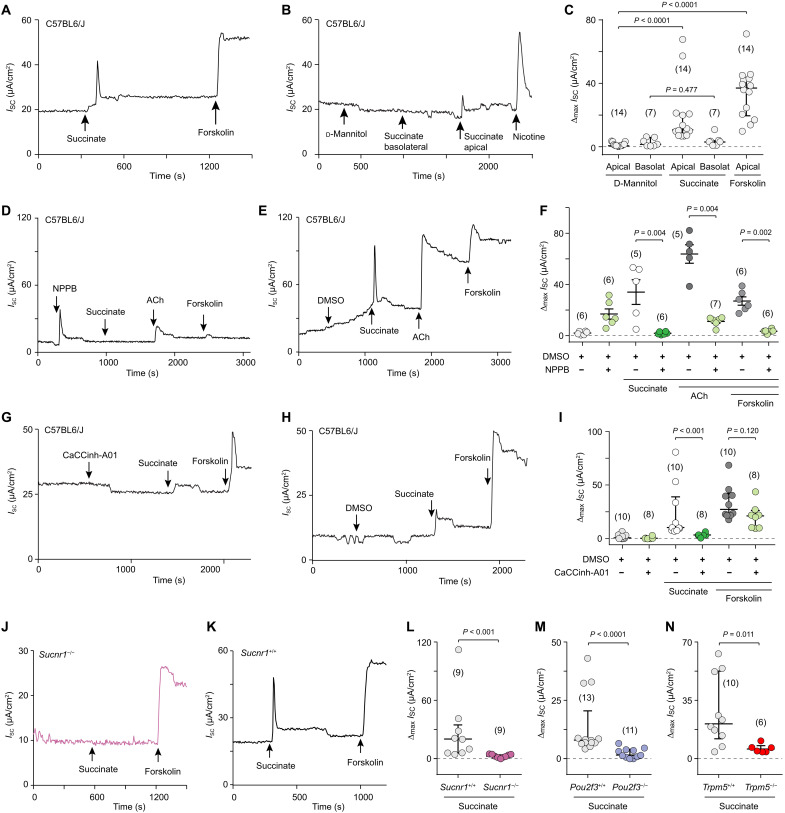
Succinate induces transepithelial Cl^−^ flux through brush cells. Representative recordings of transepithelial short-circuit current (*I*_SC_) in an opened mouse trachea mounted in an Ussing chamber and exposed to succinate (1 mM) (**A**, **B**, **D**, **E**, **G**, **H**, **J**, and **K**) and group data showing maximum increases in *I*_SC_ (Δ*I*_SC_) (**C**, **F**, **I**, and **L** to **N**). The adenylate cyclase activator forskolin [10 μM, (A) and (C) to (H)], nicotine [100 μM, (B)], and acetylcholine (ACh) [100 μM, (D) to (F)], all applied apically, served as controls for vitality of the preparation [(A) to (K)] and efficacy of Cl^−^ channel inhibition [(D) to (F)]. d-Mannitol (1 mM) served as reference with same osmolarity as succinate. Vehicle control for experiments with the Cl^−^ channel inhibitor NPPB (100 μM) [(D) to (F)] and the CaCC inhibitor CaCCinh-A01 (20 μM) [(G) to (I)] was DMSO (500 μM). Controls in experiments with gene-deficient mice (−/−) were respective littermate wild-type mice (+/+) [(J) to (N)]. Representative recordings to (M) and (N) are shown in fig. S10 (J to M). Data, median ± interquartile range (IQR), number of tracheae is given in parentheses; Mann-Whitney test. Data are from eight independent experiments in (C), from four in (F) and (M), from three in (I) and (N), and from seven in (L).

Succinate (1 mM) induced a sharp peak in *I*_SC_ when applied to the luminal (apical) but not to the basolateral side of the epithelium (Fig. 7, A to C). Water content of the periciliary fluid is balanced mainly by Na^+^ reabsorption through Na^+^ channels in the apical membrane and a gradient generated by basolateral Na^+^/K^+^-ATPase and by Cl^−^ secretion through apically located Cl^−^ channels with the intracellular Cl^−^ pool being fueled by basolateral Na^+^/K^+^/Cl^−^ cotransporters (*47*, *48*). The succinate-induced current was insensitive to the Na^+^ channel blocker amiloride (100 μM) (fig. S10, A to C) but was effectively suppressed by the nonselective Cl^−^ channel inhibitor NPPB (100 μM) (Fig. 7, D to F), in support of our hypothesis predicting secretion. In the mouse trachea, Cl^−^ secretion is dominated by Ca^2+^-activated Cl^−^ channels (CaCC) (*49*–*52*), and the general CaCC inhibitor A01 (CaCCinh-A01, 20 μM) nearly fully suppressed the succinate-induced increase in ion flux (Fig. 7, G to I). Inhibition of transmembrane protein 16A, which accounts for most of the purinoceptor-regulated CaCC activity in the mouse trachea (*52*), by Ani9 (10 μM) did not affect the response to succinate (fig. S10, D to F). CFTR, the Cl^−^/bicarbonate channel affected in cystic fibrosis, contributed to the baseline short-circuit current in our preparation as evidenced by a drop in *I*_SC_ upon administration of its inhibitor CFTRinh172 (25 μM) but did not convey the succinate effect which remained unaffected by CFTR blockade (fig. S10, G to I).

Consistent with our Ca^2+^ imaging results and the ciliary activity measurements, experiments with respective gene-deficient mouse strains (*Sucnr1^−/−^*, *Pou2f3^−/−^*, and *Trpm5^−/−^*) revealed a critical involvement of the SUCNR1–TRPM5–brush cell pathway in Cl^−^ secretion (Fig. 7, J to N). In all of these strains, the succinate-induced increase in *I*_SC_ was specifically lost, whereas baseline tracheal epithelial resistance, baseline short-circuit current, and its increase in response to forskolin were indistinguishable from wild-type C57BL6/J mice (fig. S10, J to P).

### The secretory response requires paracrine cholinergic and long-range gap junction signaling

**Fig. 8.**
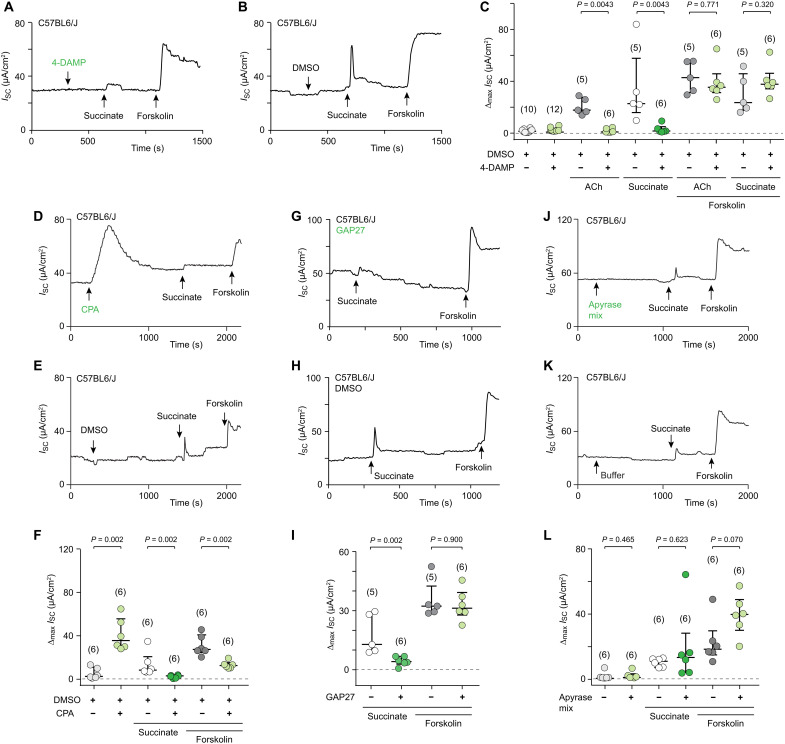
Succinate-induced Cl^−^ flux requires sequential cholinergic and gap junction signaling. (**A**, **B**, **D**, **E**, **G**, **H**, **J**, and **K**) Representative recordings of transepithelial short-circuit current (*I*_SC_) in an opened mouse trachea mounted in an Ussing chamber and exposed to succinate (1 mM), (**C**, **F**, **I**, and **L**) group data showing maximum increases in *I*_SC_ (Δ*I*_SC_). The adenylate cyclase activator forskolin (10 μM, given apically) served as vitality control throughout. Vehicle control for experiments with the muscarinic M3 receptor blocker 4-DAMP (1 μM) [(A) to (C)], the SERCA-inhibitor cyclopiazonic acid (CPA) (30 μM) [(D) to (F)], and the gap junction inhibitor GAP27 (130 μM, given 45 min before succinate) [(G) to (I)] was DMSO [(A) to (C), 1 mM; (D) to (I), 500 μM]. Apyrase was given as a mix of apyrase VI and VII, 5 U/ml each [(J) to (L)]. All inhibitors were applied both apically and basolaterally. Data are from three independent experiments in (C), from two in (F) and (I), and from five in (L). Data, median ± interquartile range (IQR), number of tracheae is given in parentheses; Mann-Whitney test.

On the basis of our previous findings, we hypothesized that cholinergic transmission constitutes a critical step also in activation of Cl^−^ secretion induced by succinate. In the mouse tracheal epithelium, both muscarinic and nicotinic AChR stimulation results in an increase in *I*_SC_ (*45*, *46*). Therefore, we first used a general nicotinic/muscarinic receptor inhibitor cocktail (25 μM mecamylamine and 25 μM atropine) which was effective in blocking ACh-induced currents (fig. S11, A, B, and E). These antagonists fully abrogated the response to succinate (fig. S11, C to E), demonstrating the principal involvement of cholinergic transmission. Consistent with the fact that *Chrm3^−/−^* mice lack a muscarine-evoked increase in short-circuit current (*45*), we found that application of 4-DAMP (1 μM) also blocked the responses to both succinate and ACh (Fig. 8, A to C, and fig. S11, F to G). The SERCA inhibitor CPA (30 μM), which suppressed Ca^2+^ wave propagation, had an initial stimulatory effect on *I*_SC_, consistent with the transient rise in [Ca^2+^]_i_ it produces (*31*), and inhibited the succinate- and, to a lesser degree, the forskolin-induced increase in *I*_SC_ (Fig. 8, D to F). We next tested the effect of gap junction blockade by GAP27 (130 μM) on ion secretory responses. This intervention did not interfere with the effect exerted by forskolin, which directly stimulates adenylate cyclase in all epithelial cells. However, it did prevent an increase in *I*_SC_ upon stimulation with succinate (Fig. 8, G to I). Carbenoxolone (100 μM), a less specific gap junction inhibitor, not only inhibited succinate-induced currents but also interfered, to some extent, with the response to ATP and had a strong activating effect when given alone (fig. S12, A to C). Last, we addressed the potential role of ATP release and P2 receptors, but neither apyrase treatment, nor the broad P2 inhibitor PPADS (100 μM), nor specific P2X4/P2X7 inhibition by 5-BDBD (10 μM) and A438079 (20 μM), nor the P2Y2 inhibitor ARC-118925XX (10 μM) suppressed the succinate-induced effect on *I*_SC_ (Fig. 8, J to L, and fig. S12, D to O). Notably, responses to ATP were largely reduced by P2X4/7 inhibition but not by the broad inhibitor PPADS (fig. S12, D to L), consistent with an earlier notion that ATP activity on mouse P2X4 expressed in *Xenopus* oocytes is not reduced but even augmented by PPADS (*53*). Such a paradoxical effect may also underlie the slight increase in succinate-induced currents in the presence of PPADS (fig. S12, D to F).

Together, these experiments validate the model that brush cells serve as a central hub after sensing luminal succinate to orchestrate a defense program in the trachea that includes enhanced mucociliary clearance and Cl^−^ secretion driven by a Ca^2+^ wave that propagates via sequential paracrine cholinergic and long-range gap junction signaling (fig. S13).

## DISCUSSION

This study identified SUCNR1 as a tracheal brush cell receptor triggering sequential cholinergic and connexon-based signaling, which results in a radially spreading long-range Ca^2+^ wave that drives overall stimulation of ciliary activity and Cl^−^ secretion. Since only apical but not basolateral application of the SUCNR1 ligand succinate induced transepithelial ion flux, this sensor is specifically designed to detect intraluminal succinate concentration which qualifies it as a monitor of danger situations. Succinate levels in the airway lumen are known to be elevated during bacterial infections (*19*, *54*), reaching concentrations that are sufficient to evoke the cilioactivating and secretory responses reported here. In contrast to previously identified activators of tracheal brush cells such as quorum-sensing molecules from *Pseudomonas aeruginosa* (*1*, *7*) and virulence-associated formylated bacterial signal peptides produced by distinct lung pathogens (*8*), succinate is not produced by bacterial pathogens settling in the airways. In the course of bacterial infection, classically activated or M1 macrophages accumulate mitochondrial succinate, which is then released into the cytoplasm and, lastly, secreted in considerable amounts, reaching >10 mM in the bronchoalveolar lavage fluid in mice infected with *P. aeruginosa* (*19*). In this scenario, succinate produced by the host even fuels infection as it is the preferred carbon source for *P. aeruginosa* (*55*).

In the small intestine, in contrast, it is microbiota-derived succinate that is sensed by the tuft cells, the intestinal counterpart of airway brush cells. Both the helminth *Nippostrongylus brasiliensis* and a tritrichomad protist secrete succinate as a metabolite which stimulates tuft cells through SUCNR1 to induce a multifaceted type 2 immune response (*16*, *17*, *30*, *56*). Before settling in the small intestine, larvae of *N. brasiliensis*, like those of many other helminths, migrate to the lung (*57*) where they induce a strong type 2 immune response (*58*, *59*). Succinate levels have not been quantified under these conditions, but the larvae are already equipped with all enzymes of the Krebs cycle enabling the formation of succinate from isocitrate (*60*). Thus, helminth larvae on lung transit may also represent a potential natural source of succinate monitored by airway brush cells.

The receptors addressed by previously recognized activators of tracheal brush cells all remained unidentified, but their downstream signaling shares key components of the SUCNR1 pathway identified in this study. They all trigger ACh release from brush cells dependent on PLCβ2 (*8*) and TRPM5 (*1*, *7*, *8*), potentially enabling brush cells to integrate diverse signals into a common defense program. This program does include not only effects in the immediate vicinity, e.g., feedback through regulatory muscarinic autoreceptors or excitation of closely attached sensory nerve fibers (*1*, *2*), but also overall activation of ciliated cells resulting in a net increase in particle clearance (*7*, *8*). Given the paucity of brush cells and the long distances to be bridged to induce such a global mucosal response, it remained unclear whether brush cell–derived ACh reaches distant cells at sufficiently high concentrations to directly stimulate ciliary activity. An entirely different communication pathway has been proposed in human sinonasal mucosa. Here, the bitter compound denatonium evokes a localized Ca^2+^ signal in discrete cells—presumably solitary chemosensory cells, the sinonasal counterpart of tracheal brush cells—that subsequently spreads to the surrounding cells depending on intact gap junction communication (*6*). Using an intact tracheal preparation allowing unequivocal identification of brush cells combined with monitoring of Ca^2+^ signals at a high spatiotemporal resolution, we here show that these models are not principally exclusive. Instead, both communication pathways operate sequentially: Initial circumscribed paracrine cholinergic signaling triggers a long-range Ca^2+^ wave that drives distant cellular reactions. Propagation of this wave and cellular effector responses required GAP27-sensitive connexins but were insensitive to Cx43 hemichannel blockade, to apyrase, and to P2 receptor inhibition, strongly indicating gap junction signaling.

The Ca^2+^ wave triggers concomitant stimulation of ciliary activity and Cl^−^ secretion, which are synergistic in promoting mucociliary clearance, because Cl^−^ secretion is a prerequisite for mucus hydration and the formation of a sufficiently high layer of low-viscous periciliary fluid needed for effective ciliary stroke. An additional contribution of basolateral Ca^2+^−dependent K^+^ channels to the Ca^2+^-dependent prosecretory response cannot be excluded. Very recently, resorption rather than secretion of Cl^−^ has been reported in the mouse trachea exposed to the bitter compound and brush cell activator denatonium (*61*), which would rather impede than support mucociliary clearance. This denatonium response differed also in several further aspects from that evoked by succinate, e.g., in being connected to CFTR and insensitivity to inhibition of muscarinic receptors and gap junctions/hemichannels (*61*). This matches with previous findings in rat tracheal slice preparations that denatonium directly activates 40% of all epithelial cells (*62*), underlining the need of selective stimuli to clarify the role of brush cells in epithelial defense mechanisms. In summary, the SUCNR1–brush cell–ACh–gap junction pathway translates elevated succinate in the airway lining fluid, indicative of the presence of pathogenic microbiota, into a critical defense program essential for the removal of luminal content and the avoidance of airway diseases.

We see two limitations of this study. First, although there is ample evidence that connexin-based signaling in the epithelium operates through gap junctions, we cannot fully rule out release of a yet unknown mediator other than ATP being released through hemichannels formed by GAP27-binding connexins other than Cx43 and acting on other receptor classes. Second, the role of this pathway in human airway physiology still needs to be elucidated. Up to now, preliminary evidence has been presented for activation of human sinonasal solitary chemosensory cells, an upper airway variant of tuft cells, by succinate (*63*), and single-cell RNA sequencing data revealed a baseline expression of SUCNR1 in brush (tuft) cells in human intrapulmonary airways (*64*), which was markedly up-regulated in allergic conditions (*65*). Thus, SUCNR1 also might play a role in human brush cells, particularly under pathological conditions.

## MATERIALS AND METHODS

### Study design

The aim of this study was to identify a ligand-receptor pair activating brush cells in the mouse trachea to help analyze the spread of activity from rare brush cells to both ciliated and secretory cells over the mucosal surface with its effects on ciliary activity and transepithelial ion transport. The events under investigation require intercellular communication in the natural context, but not systemic interorgan communication, so that analyses were done at freshly explanted tracheae. We hypothesized that succinate could function as a potential brush cell stimulator and that ACh would be released as the initial messenger upon stimulation. We used spatiotemporal Ca^2+^ imaging to monitor initial excitation of brush cells and spread of information within the epithelial layer and measured particle transport on the mucosal surface and transepithelial currents as readouts for ciliary activity and ion transport, respectively. The underlying cellular and molecular mechanisms were analyzed using appropriate genetic models and pharmacological inhibitors.

### Mice

Adult mice (older than 6 weeks) of either sex were used. Ca^2+^ imaging experiments were conducted on mice kept in a specific pathogen–free (SPF) facility with 12-hour light/12-hour dark cycle with food (Ssniff feed containing 9% fat, 24% protein, and 67% carbohydrate) and water ad libitum. Mice taken for all other experiments were kept in a SPF facility with 10-hour light/14-hour dark cycle with food (1320 Maintenance diet for rats and mice, Altromin, containing 11% fat, 24% protein, and 65% carbohydrate) and water ad libitum. Mice were housed in individually ventilated cages containing enrichment (nesting, bedding, and other material). We used the following mouse strains: C57BL6/J (Charles River), *Trpm5*-IRES-*Cre* (*66*), B6;129S-Gt(ROSA)26Sortm95.1(CAG-GCaMP6f)Hze/J (RRID:IMSR_JAX:024105, referred to as R26-GCaMP6f or Ai95D mice) (*67*), and B6.Cg-Gt(ROSA)26Sortm14(CAG-tdTomato)Hze/J (JR # 007914, referred to as R26-tdTomato mice) (*68*). We crossed *Trpm5*-IRES-Cre mice with R26-GCaMP6f or R26-tdTomato reporter mice resulting in a strain in which all TRPM5^+^ cells are identifiable through their green fluorescence (referred to as *Trpm5*-GCaMP6f mice) or their red fluorescence (referred to as *Trpm5*-tdTomato mice). These mice were also crossed with the global *Plcb2^−/−^* mice [(*69*); provided by D. Wu, Yale University, New Haven, CT, USA], resulting in a strain in which all cells are deficient for *Plcb2*, and all TRPM5^+^ cells are identifiable through their green or red fluorescence (referred to as *Trpm5*-GCaMP6f;*Plcb2*^**−/−**^ or *Trpm5*-tdTomato;*Plcb2*^**−/−**^). *Trpm5*-GCaMP6f, *Trpm5*-tdTomato, *Trpm5*-GCaMP6f;*Plcb2*^**−/−**^, or *Trpm5*-tdTomato;*Plcb2*^**−/−**^ mice were heterozygous for *Cre* and GCaMP6f or tdTomato, respectively. The TRPM5-GFP/*Trpm5*^−/−^ mice in the Ca^2+^ imaging experiments are a cross between transgenic *Trpm5*-GFP and global *Trpm5^−/−^* mice (*27*, *29*, *70*, *71*) and provided by D. Restrepo (University of Colorado, Aurora, CO, USA). *Trpm5*-eGFP mice [Tg(Trpm5-EGFP)^#Sdmk^] (*26*) used for cell isolation and sorting were provided by R. F. Margolskee (Monell Chemical Senses Center, Philadelphia, PA, USA). *Trpm5^−/−^* mice used in PTS and Ussing chamber experiments were obtained from The Jackson Laboratory (stock no. 005848; *Trpm5^tm1Dgen^*, 129P2/OlaHsd), where they had been backcrossed at least six generations onto the C57BL/6J background. *Pou2f3^tm1Abek^* (*Pou2f3^−/−^*) mice were characterized previously (*48*, *72*), heterozygous or homozygous wild-type littermates (+/− or +/+) served as controls. Cell type–specific deletion of *Chat* in brush cells was reached by breeding B6;129-*Chat^tm1Jrs^*/J (*Chat^fl^*) purchased from The Jackson Laboratory (RRID:IMSR_JAX:024105) with B6;D2-Tg(*Avil-cre*)1Phep/Cnrm (*Avil^cre^*) mice (*73*), as described previously (*8*).

Mice except those for Ca^2+^ imaging were killed by inhalation of an overdose of 5% isoflurane (Abbott, Wiesbaden, Germany) and exsanguination through abdominal blood vessels. Animal care and experimental procedures were performed in accordance with the guidelines established by the German Animal Welfare Act, European Communities Council Directive 2010/63/EU, the institutional ethical and animal welfare guidelines of the Saarland University (approval number of the Institutional Animal Care and Use Committee: CIPMM-2.2.4.1.1), the Justus Liebig University Giessen (571_M, 641_M, 793_M and 741_M), and the Philipps-University Marburg (Ex-02-2021). The number of animals used is a minimum necessary to provide adequate data to test the hypotheses of this project. We minimized the number of animals required by the animal welfare committees wherever possible.

### Generation of the global *Sucnr1^−/−^* mouse line

The CRISPR-RNAs, trans-activating CRISPR-RNA, repair templates, duplex buffer, and Cas9 protein were purchased from Integrated DNA Technologies (IDT) Integrated DNA technologies. Two custom crRNAs were designed by the CRISPR guide tool (IDT Integrated DNA technologies). The crRNAs were located to cut 144 bp upstream (5′-GGCATGGACCTTCAATACGAGGG-3′) and 489 bp downstream crRNA (5′-CATTCCAACTGACGAACACCTGG-3′) of exon 2 of the *Sucnr1* gene. As repair template for homolog recombination and loxp integration, two single-stranded oligodeoxynucleotide DNA constructs were designed (upstream: 5′-tgtagataggttgtcctggaactcatagagatctgcttgtctctggttctccagccctcgATAACTTCGTATAATGTATGCTATACGAAGTTATtattgaaggtccatgccatcaagtatgacaagcacattctttatcaaccctccccagtta3′ and downstream 5′gtcttaactattgctcttgcagaggtttaggctcagttctcggcattccaactgacgaacATAACTTCGTATAATGTATGCTATACGAAGTTATacctggtaacttcataacagttcctatcattccagctccatggagttagaggccccagtc 3′). According to the manufacturer’s protocol, the crRNA and tracrRNA (5 μl of a 100 μM stock of each RNA in duplex buffer) were annealed to form the crRNA:tracrRNA complex and stored at −80°C until the day of electroporation. One hour before electroporation in a final volume of 40 μl Opti-MEM (Gibco, Thermo Fisher Scientific), 4 μg of each crRNA:tracrRNA complex with 4 μg Cas9 protein and 4.6 μg ssODN DNA was mixed, and the resulting RNP complex was used for electroporation.

For electroporation, 5 μl of the RNP-complex and approximately 40 fertilized eggs were used in an in-house self-made platin plate electroporation slide with a 1 mm gap (similar to CUY501P1-1.5 from Nepa). For electroporation, a NEPA21 Super Electroporator (Nepagene) was used with the following settings: poring pulse of 40 V; pulse length: 3.5 ms; pulse interval: 50 ms; number of pulses: 4; decay: 10%; polarity: + directly followed by a transfer pulse of 5 V; pulse length: 50 ms; pulse interval: 50 ms; number of pulses: 5; decay: 40%; polarity: +/−.

When screening the newborns for integration of *loxp* sites, we got one pup without integration of the ssODN and *loxp* site but a deletion of *Sucnr1* exon 2. The global *Sucnr1^−/−^* mice were confirmed by sequencing (*CTCTGGTTCTCCAGCCCTCG*CTTCATAACAGTTCCTATCA). The deletion ranges from 144 bp upstream of exon2 up to 498 bp downstream of exon 2. For genotyping, two PCRs are performed. The wild-type allele was amplified with one primer upstream of exon 2 and one primer in the targeted region (5′ gatctgcttgtctctggttctc; 3′ cctctgttgccaaccaattct) resulting in a 215-bp PCR product for the wild-type allele and no product for the knockout allele. For the knockout, allele a primer combination was used overspanning exon 2 (5′ gatctgcttgtctctggttctc; 3′ tatgtgtgtgcctgcttgattgtg) resulting in a 2295-bp product for the wild-type allele and a 142-bp product for the knockout allele.

### Epithelial cell isolation and fluorescence-activated cell sorting

Fluorescence-activated cell sorting (FACS) experiments were conducted twice, one experiment using five and the other using three *Trpm5*-eGFP mice. Tracheae were dissected, freed from surrounding tissue, opened by cutting the trachealis muscle longitudinally, and digested using a modification of a protocol previously described by Ualiyeva *et al.* (*74*). Specimens were transferred into a digestion solution containing 16 mg of dispase II (Roche, catalog no. 4942078001) and 1 μl of 25 mM deoxyribonuclease I (DNase I; Invitrogen, catalog no. 18068015) in 1 ml of phosphate-buffered saline (PBS) per trachea and incubated for 40 min at room temperature on a shaker (200 rpm). Digestion was stopped by adding 750 μl of ice cold Dulbecco’s minimum essential medium supplemented with 5% of fetal calf serum (FCS; Thermo Fisher, catalog no. 10082147). The tracheae were placed in a petri dish, and the epithelium was removed mechanically using a scalpel. The trachea was rinsed with 750 μl of Tyrode I buffer containing 140 mM NaCl, 5 mM KCl, 1 mM MgCl_2_, 10 mM Hepes, 10 mM glucose, 1 mM sodium pyruvate, papain (26 U/ml; Sigma-Aldrich; #P4762), and l-cysteine (10 μl/ml; Sigma-Aldrich, catalog no. 14495). The epithelium and the Tyrode I buffer were transferred into a 2-ml tube and incubated for 30 min at 37°C on a shaker (200 rpm) in the dark. To stop the second digestion, 750 μl of Tyrode II solution, containing 140 mM NaCl, 5 mM KCl, 1 mM MgCl_2_, 1 mM CaCl_2_, 10 mM Hepes, 10 mM glucose, 1 mM pyruvic acid, and leupeptin (5 mg/ml; Sigma-Aldrich, CAS no. 103476-89-7), was added to each epithelial sample, and the tube was vortexed. For further separation of epithelial cells, the fluid was triturated 10 times through a syringe attached to 18G needle and again 10 times through a 21G needle. The homogenates processed in one experiment were filtered through a 100-μm cell strainer and collected in a 50-ml Falcon tube, to which 30 ml of cold FACS buffer (Miltenyi, catalog no. 130-092-747) complemented with 2% FCS was added. The cells were washed by centrifugation for 10 min at 700 rpm at 4°C, the supernatant was discharged, and the pellet was dissolved in FACS buffer. This step was repeated and the pellet was lastly reconstituted in 100 μl of FACS buffer per mouse. To block unspecific binding, anti-mouse CD16/32 blocking antibody (1:100; Miltenyi, catalog no. 130-092-574) was added and incubated for 15 min on ice in the dark. Antibody and live-and-dead staining was done for 15 min at 4°C protected from light with rat monoclonal antibodies against EpCAM (anti-mouse CD326, PE-Vio 770, Miltenyi; 1:50; RRID: AB_2657522) and CD45 (anti-mouse CD45, VioBlue, Miltenyi; 1:50; RRID: AB_2751586) and the FVS700 live-and-dead staining kit (1:1000; BD Biosciences, AB_2869637). Cells were washed by adding 2 ml of FACS buffer and subsequent centrifugation at 700 rpm for 10 min at 4°C, and cells were resuspended in 2 ml of FACS buffer and again washed. The resuspended cells were separated by FACS immediately. Samples were FSC-A/SSC-A gated to exclude debris and FSC-H/FSC-A gated to select single cells. Dead cells were excluded by staining with FVS700. Brush cells were obtained by gating for CD45^low^, EpCAM^high^, and GFP^pos^; epithelial cells were obtained by gating for CD45^neg^, EpCAM^high^, and GFP^neg^; immune cells were obtained by gating for CD45^high^, EpCAM^hneg^, and GFP^neg^, using a FACS Aria III (BD Biosciences) and FACS Diva Software (BD Biosciences). Gating strategy was visualized using FlowJo v.10.7.1.

### RT-PCR and quantitative PCR

For samples obtained by FACS, cells were centrifuged, and mRNA was isolated immediately using the RNeasy Micro Kit (Qiagen, catalog no. 74004) according to the manufacturer’s protocol. Contaminating DNA was eliminated by on-column digestion using DNase Set (Qiagen, catalog no. 79254). SuperScript II (Invitrogen, catalog no. 18064022) was used for cDNA synthesis, priming was done using a 1:1 mixture of random hexamers (Invitrogen, catalog no. SO142) and oligo dTs (MWG-Biotech). RNA from trachea, tongue, kidney, and white fat was isolated using the RNeasy Mini Kit (Qiagen, catalog no. 74106). Contaminating DNA was eliminated by prior DNA digestion (DNase I, Invitrogen, catalog no. 18068015). Synthesis of cDNA was done using SuperScript II according to the manufacturer’s protocol. Tracheal epithelium was abraded mechanically using sterilized cotton swabs dipped in lysis buffer, and RNA extraction and cDNA synthesis were done as described for FACS samples. Samples without reverse transcriptase and PCR mix without cDNA served as negative control for PCR. PCR was performed by adding 1 μl of cDNA, 0.6 μl of each primer pair (10 pM, MWG-Biotech; primers are provided in table S1), 2.5 μl of 10× PCR buffer II (Thermo Fisher, catalog no. N8080259), 1.5 μl of MgCl_2_ (Thermo Fisher Scientific, catalog no. N8080259), 0.5 μl of deoxynucleotides (Qiagen, catalog no. 201912), 0.5 μl of AmpliTaqGold polymerase (5 U/ml; Thermo Fisher, catalog no. N8080259), and 18.5 μl of H_2_O. Cycling conditions were 5 min at 95°C, followed by 40 cycles of 45 s at 95°C, 30 s at 60°C, 45 s at 72°C, and a final extension at 72°C for 10 min.

Quantitative real-time PCR was performed with the same primers using the I-Cycler IQ detection system (Bio-Rad, Munich, Germany) in combination with the IQ SYBR Green Real-Time PCR Supermix (Bio-Rad, catalog no. 1708882). The PCR conditions included initial denaturation in 1 cycle of 3 min at 95°C followed by 45 cycles of 20 s at 95°C, 20 s at 60°C, and 20 s at 72°C. The relative expressions were calculated as: 2^-(ΔCT)^ × 1/mean control 2^-(ΔCT)^, where ΔCT is calculated as: ΔCT = CT_GOI_ − CT_HKG_ (GOI, gene of interest; HKG, housekeeping gene, here β*-actin*). Samples were measured in duplicate and the mean was taken for further evaluation. The PCR products were separated by electrophoresis on a 2% tris-acetate-EDTA agarose gel with ethidium bromide.

### In silico analysis of single-cell RNA sequencing data

Previously published gene expression data (*21*, *35*) (datasets GSE103354 and GSE102580) of tracheal epithelial cells from C57BL/6J mice were downloaded from National Center for Biotechnology Information Gene Expression Omnibus. The data were reanalyzed, and reclustering was done using the Seurat R package (version 2.3.4) (*75*). Linear dimensionality reduction was performed using principal components analysis. UMAP (Uniform Manifold Approximation and Projection) was used for nonlinear dimension reduction (*76*). For visualization and interpretation of clustering, cells were presented in a 2D UMAP plot, and based on the expression of typical marker genes, clusters were identified and annotated accordingly.

### Confocal Ca^2+^ imaging of TRPM5^+^ cells in tracheal whole mounts

Mice (6 to 23 weeks old, either sex) were anesthetized by intraperitoneal injection with 165 mg/kg body weight ketamine (Pharmacia GmbH, Berlin, Germany) and 11 mg/kg body weight xylazine (Bayer Health Care, Leverkusen, Germany) according to institutional guidelines. The trachea was dissected from the esophagus, then cut below the larynx and above the bifurcation, and transferred to a solution (preparation solution) containing 140 mM NaCl, 5 mM KCl, 1 mM MgCl_2_, 2 mM CaCl_2_, and 10 mM Hepes, adjusted to pH 7.4 (NaOH). The respiratory epithelium was exposed by cutting the trachea along the trachealis muscle. The trachea of a mouse was divided into two halves (along one of the tracheal cartilage rings), and each half was used for one independent experiment. Intracellular Ca^2+^ was monitored with either GCaMP6f expressed in TRPM5^+^ cells of *Trpm5*-GCaMP6f mice (*8*, *77*) or the Ca^2+^ indicator Cal520-AM loaded into tracheal epithelial cells using a similar technique as previously described (*77*–*79*) with adjusted dimethyl sulfoxide (DMSO) and Pluronic F-127 content. Cal520-AM (AAT-Bioquest, catalog no. 21130) was dissolved in a solution of DMSO and freshly prepared 20% Pluronic F-127 in DMSO, then further diluted in preparation solution (see above), and briefly sonicated. The tracheal epithelium was subsequently incubated in the Cal520-AM loading solution having a final concentration of 9 μM Cal520-AM, 0.01% Pluronic F127, and 0.0025% DMSO for 60 min at room temperature. In *Trpm5*-GFP^−/−^ mice, baseline green fluorescence was relatively high within the TRPM5^+^ cells due to concomitant GFP and Cal520 fluorescence, but fluorescence saturation was not reached so that ligand-evoked responses could be measured. Before beginning the experiments, the tracheal epithelium was perfused for 10 min with dye-free extracellular bath solution containing 136.5 mM NaCl, 5.6 mM KCl, 2.2 mM CaCl_2_, 1 mM MgCl_2_, 10 mM Hepes, adjusted to pH 7.4 (NaOH), and 290 mOsm (~10 mM glucose). The tracheal epithelium was secured in a laminar flow chamber (Luigs & Neumann) using a harp and superfused continuously with the extracellular bath solution at a rate of ~10 μl/s.

The Leica TCS SP5 confocal microscope was equipped with a 20× 1.0 water-immersion objective (HCS APO L). Excitation wavelength for GCaMP6 or Cal520 was 488 nm, and emitted fluorescence was collected between 500 and 540 nm. The Stokes shift of tdTomato permitted multicolored analysis in tdTomato-labeled TRPM5^+^ cells in *Trpm5*-tdTomato mice using excitation wavelength of 543 nm. Emitted fluorescence for tdTomato was collected between 580 and 640 nm. All scanning head settings were kept constant during each experiment. Optical sections were 4- to 9-μm thick. High-resolution scans (512 × 512 pixels per frame) were made using the confocal zoom up to maximally 6.0. Images were acquired every 0.52, 0.74, or 1.29 s. The preparation was inspected for health and ciliary beating before and after confocal imaging using the charge-coupled device camera (Leica DFC360Fx) on the Leica confocal scanning microscope.

The following preestablished criteria for stimulus-induced Ca^2+^ responses were applied: (i) A response was defined as a stimulus-dependent deviation of either GCaMP6f or Cal520 fluorescence signal that exceeded twice the SD of the mean of the baseline fluorescence noise. (ii) Cells showing a response to control buffer were excluded from analysis. (iii) A response had to occur within 2 min after stimulus application. In time series experiments, ligand application was repeated to confirm the repeatability of a given Ca^2+^ response. GCaMP6f or Cal520 fluorescence changes of individual cells are expressed as relative fluorescence changes, i.e., Δ*F*/*F* (*F* was the average during control stimulation with extracellular bath solution). Cal520 fluorescence waves of the tracheal epithelium are expressed as Δ*F*/*F* density which is the Δ*F*/*F* of the standardized FOV divided by area of the standard FOV (123.02 μm by 123.02 μm = 15133.9 μm^2^). Optical sections (*z* axis) of all recordings were kept constant. To capture the spatiotemporal properties of Cal520 fluorescence waves quantitatively, we analyzed Δ*F*/*F* density values per standard FOV as a function of time. The peak Δ*F*/*F* density values were compared either between control and pharmacological treatment or between wild-type and knockout mice. One to six Δ*F*/*F* densities per standard FOV were averaged in an independent trachea experiment. Images were analyzed using Fiji/ImageJ (National Institutes of Health), Igor Pro (Wavemetrics), and Originlab (Origin) software. Through the Igor Pro software package, user-defined functions in combination with an iterative Levenberg-Marquardt nonlinear, least-squares fitting routine were applied to the data. Dose-response curves were fitted by the equation

$$f(x)=E_{\min}+(E_{\max}-E_{\min})/\{1+{\left[{\mathrm{EC}}_{50}/x\right]}^{n}\}$$

where *x* is the drug concentration, *E*_min_ is the baseline response, *E*_max_ is the maximal response at saturating concentrations, and EC_50_ is the drug concentration that produces 50% of the maximal response with slope *n* being the Hill coefficient of the sigmoid curve. Images of the mean peak Ca^2+^ waves are mean fluorescence intensities (18 frames, 512 × 512 pixels, Δ*t* = 0.74 s, time frame = 13.32 s) at rest and during ligand-evoked peak responses.

### Chemicals for Ca^2+^ imaging

The tracheal epithelium was stimulated successively using bath application. Chemostimuli for Ca^2+^ imaging were prepared fresh daily and diluted in extracellular solution giving the following final concentrations: succinate (1 mM, CAS no. 6106-21-4), ATP (20 μM, CAS no. 74804-12-9), cycloheximide (10 mM, CAS no. 66-81-9), and denatonium benzoate (10 mM, CAS no. 3734-33-6). Pharmacological treatments were applied either directly into the bath chamber or via gravity flow into the bath. Atropine (10 μM, CAS no. 5908-99-6) and 4-DAMP (1 μM, CAS no. 1952-15-4) were used to inhibit muscarinic AChRs; mecamylamine (100 μM, CAS no. 826–39-1) to inhibit nicotinic AChRs. The involvement of connexins, or gap junctions, was examined with GAP27 (130, 200, or 300 μM, Tocris Bioscience, CAS no. 198284-64-9) and the Cx43 hemichannel blocker TAT-GAP19 (100 μM, CAS no. 1507930-54-2). The different concentrations of GAP27 did not affect the tracheal epithelium differently. The involvement of ATP or ADP was examined using the enzymes apyrase VI (10 U/ml) or a mix of apyrase VI and apyrase VII (5 U/ml each, CAS no. 9000-95-7). The two apyrases have different enzyme activities for ATP and ADP (*41*). Purinergic receptor involvement was assessed using the broad P2X and P2Y antagonist PPADS (100 μM, CAS no. 192575-19-2). The impact of intracellular Ca^2+^ stores was examined with CPA (30 μM, CAS no. 18172-33-3). Drugs were prepared as stock solutions in DMSO or distilled water and diluted to the final concentration in external bath solution (see the “Confocal Ca2^+^ imaging of TRPM5^+^ cells in tracheal whole mounts” section) for bath application. Treatment time varied between drugs: atropine, mecamylamine, or 4-DAMP: 10 min; GAP27: 30 to 45 min; TAT-GAP19: 30 min; apyrase VI: 10 min; apyrase mix: 10 min; PPADS: 10 min; CPA: 15 to 20 min. Final DMSO concentrations (<0.1%, vol/vol) were tested in control solutions and had no effects. All chemicals were obtained from Merck (previously Sigma/Aldrich) if not otherwise stated.

### Post hoc immunostaining

Post hoc immunostaining of tracheal epithelium after Ca^2+^ imaging was performed at 20°C directly in the recording chamber of the Leica confocal imaging setup (*80*). Following Ca^2+^ imaging of tracheal epithelium from Trpm5-tdTomato mice, the tissue in three experiments was fixed using PBS containing 4% paraformaldehyde (10 min), washed with PBS (10 min), and then permeabilized for 10 min in blocking solution (PBS containing 0.3% Triton X-100 and 4% horse serum). Next, the tissue was incubated with rabbit anti-beta IV tubulin (1:400; Abcam, ab179509, RRID:AB_2716759) for 30 min at 20°C, then washed 1× with PBS (5 min), and incubated in secondary antibody (1:500; Alexa Fluor 555 donkey anti-rabbit immunoglobulin, Invitrogen A-31572, RRID:AB_162543) for 30 min at 20°C. After a final wash with PBS for 5 min, the tissue was imaged for anti-beta IV tubulin staining. Similar to a previous protocol (*81*), immunofluorescence images were then digitally superimposed onto the Δ*F*/*F* Ca^2+^ response and tdTomato maps using careful alignment of characteristic landmarks, which were made by the pattern of tdTomato-labeled TRPM5 expressing cells and also visible in the immunofluorescence images.

### Measurement of PTS

A modified version of the method previously described by Klein *et al.* (*42*) was used. Tracheae were explanted, transferred in a delta T-dish coated with a thin layer of Sylgard polymer (Dow Corning), and submerged in 1.5 ml of Hepes buffer containing 5.6 mM KCl, 140 mM NaCl, 1 mM MgCl_2_, 2.2 mM CaCl_2_, 5 mM d-glucose, and 10 mM Hepes (pH 7.4). Temperature (31°C) was controlled during the experiments using a Bioptechs Delta T micro-environmental control system (Bioptechs). The trachealis muscle was cut, and the trachea was fixed flat with needles on the bottom of the dish with the mucosal side facing up. Before measurements (videos taken), polysterene dynabeads (1.5 μl) (Invitrogen, catalog no. 10003D) were added onto the tracheal surface. The first measurement was done after 30 min of equilibration time, followed by measurements every 5 min until 55 min after start and then every 2 min. Stimulation started after 55 or 59 min. Before each measurement, buffer and dynabeads were well mixed in the dish by pipetting. Videos consisting of 200 images (640351 pixels; 12 bit; 1 image per 84 ms) were taken using a UMPLFL20xW/0.5 water immersion objective (Olympus) and a SMX-150 M (EHD imaging GmbH) camera. Movement of dynabeads was tracked and visualized by using Image-Pro Plus (MediaCybernetics) software, and the average speed of all tracked particles over a 15 s time period was calculated. To quantify the effect of the applied substance on PTS, the maximum change in PTS within 10 min after application was calculated in %. ATP (100 mM, Sigma-Aldrich, CAS no. 102047-34-7) was added 10 min before the end of each experiment and served as a viability control. In experiments with apyrase and PPADS, which potentially interfere with the ATP effect, forskolin (10 μM, Sigma-Aldrich, CAS no. 66575-29-9) served as an additional control stimulus. Tracheae which did not respond to at least one stimulus with an increase in PTS of >25% were excluded from the data analysis (preestablished exclusion criterion). Each trachea was analyzed in an independent experiment. Succinate (Sigma-Aldrich, CAS no. 6106-21-4), atropine (10 μM, Sigma-Aldrich, CAS no. 5908-99-6), apyrase grades VI and VII (each 5 U/ml, Sigma-Aldrich, CAS no. 9000-95-7), and PPADS (100 μM, Tocris, CAS # 192575-19-2) were dissolved in the same Hepes buffer as used for the measurements. CPA (30 μM, Sigma-Aldrich, CAS no. 18172-33-3), 5-BDBD (10 μM, Tocris, CAS no. 768404-03-1), A-438079 (20 μM, Tocris, CAS no. 899507-36-9), and forskolin were dissolved in DMSO. GAP27 (130 μM, Tocris, CAS no. 198284-64-9) was dissolved in DMSO and added 45 min before the application of succinate. Experiments with *Sucnr1*-, *Plcb2*-, and *Trpm5*-deficient mice were performed blinded to the genotype.

### Measurement of CBF

Tracheae were prepared as described for PTS measurements, except that images were taken at higher magnification using an Olympus UMPLFL40xW/0.8 water immersion objective. Videos consisting of 1000 images with a frame rate of 105 images/s were taken every minute after 50 min equilibration time, succinate (1 mM, Sigma-Aldrich, CAS no. 6106-21-4) was added after 58 min, and lastly ATP (100 μM) was added. Mean CBF was calculated as described before (*8*, *82*, *83*). Briefly, dominating beat frequency of each ciliated cell (30 to 45 cells per video) was analyzed by fast Fourier transformation using a Graphical User Interface (GUI) for MATLAB R2016b, which was programmed by M. Pieper and P. König (University of Lübeck, Germany). CBF was visualized by creating false color-coding pictures using the MATLAB GUI. Tracheae which did not respond to at least one stimulus with an increase in CBF of >20% and cells with a baseline CBF of <5 Hz were excluded from the data analysis (preestablished exclusion criteria). Each trachea was analyzed in an independent experiment.

### Aequorin Ca^2+^ assay

The assay was performed as described previously (*84*). In summary, 2 × 10^4^ human embryonic kidney–293 cells (immortalized human embryonic kidney cells, obtained from American Type Culture Collection, RRID: CVCL_0045) were seeded onto 96-well plates and transfected with pcDNA3 plasmids carrying cDNAs encoding a calcium-sensitive bioluminescent fusion protein consisting of aequorin and GFP (*85*) and a promiscuous G protein α-subunit and the human SUCNR1 (cDNA resource center, Cat-Nr: SUCNR10000) or an empty pcDNA3 plasmid (mock) using Lipofectamine 2000. After 48 hours, cells were loaded with 5 μM coelenterazine h (Promega) in Hanks’ balanced salt solution containing 1.8 mM Ca^2+^ and 10 mM glucose for 2 hours at 37°C. Measurements were performed using a luminometric plate reader (Flexstation 3, Molecular Devices). The Ca^2+^ transient was measured for 2 min, and the area under curve was calculated using SoftMaxPro software. Two independent experiments were performed for each tested condition.

### Ussing chamber recordings

Tracheae were dissected, cleaned from surrounding connective tissue, longitudinally opened by cutting the trachealis muscle, and transferred into customized Ussing chambers with a circular inlet opening of 2 mm in diameter (3.142 mm^2^) (Scientific Instruments, Simmerath, Germany). The total volume of the Ussing chamber was 6 ml, with 3 ml each in the apical and basolateral compartment. The chamber was filled with buffer solution containing 117 mM NaCl, 4.7 mM KCl, 25 mM NaHCO_3_, 1.2 mM MgSO_4_, 1.2 mM KH_2_PO_4_, 2.5 mM CaCl_2_, and 5 mM d-glucose. The buffer was continuously gassed with 5.3% CO_2_, 21% O_2_, and 73.7% N_2_, before and during each measurement the pH (7.4) was controlled. Temperature was kept constant at 37°C by a heating circulator (Julabo CORIO CD). The chamber was connected via two Ag/AgCl voltage and two Ir/Pt current electrodes to a voltage clamp (VCC6, V10; ussing-chamber.com). After an equilibration period of approximately 5 to 10 min, the spontaneously generated transmembrane voltage (*V*_t_) was clamped to 0 V. Transepithelial *I*_SC_ was continuously recorded using custom-made software (Clamp version 11.0). Transepithelial resistance (ohm/cm^2^) was measured throughout the experiment. The tracheae were equilibrated for another 20 min before the experiments started. Chemicals were applied apically if not stated otherwise in the figure legends. Up to six chambers, each with one trachea, could be processed simultaneously, so that appropriate controls (vehicle control or wild-type animals) were run in parallel to the experimental conditions in each experiment. d-Mannitol (1 mM, Sigma-Aldrich CAS no. 69-65-8) served as osmolality control for succinate (1 mM). At the end of each measurement, either forskolin (10 μM, Sigma-Aldrich, CAS no. 66575-29-9), ATP (100 μM), or nicotine (100 μM, Sigma-Aldrich, CAS no. 6019-06-3) was applied apically as a vitality control. Tracheae which did not respond to at least one stimulus with a change in *I*_SC_ > 25% were excluded from the data analysis (preestablished exclusion criterion). NPPB (100 μM, Tocris, CAS no. 107254-86-4), 4-DAMP (1 μM, Sigma-Aldrich, CAS no. 1952-15-4), CaCCinh-A01 (20 μM, Tocris, CAS no. 407587-33-1), CPA (30 μM, Sigma-Aldrich CAS no. 18172-33-3), CFTR_inh_172 (25 μM, Tocris, CAS #307510-92-5), Ani9 (10 μM, Tocris, CAS no. 356102-14-2), 5-BDBD (10 μM, Tocris CAS no. 768404-03-1), A-438079 (20 μM, Tocris, CAS no. 899507-36-9), and ARC-118925 (10 μM, Tocris, CAS no. 216657-60-2) were dissolved in DMSO. Amiloride (10 μM, Sigma-Aldrich, CAS no. 2016-88-8) and GAP27 (130 μM, Tocris, CAS no. 198284-64-9), apyrase grades VI and VII (each 5 U/ml, Sigma-Aldrich, CAS no. 9000-95-7), atropine (10 μM, Sigma-Aldrich, CAS no. 5908-99-6), mecamylamine (100 μM, Sigma-Aldrich, CAS no. 826-39-1), carbenoxolone (100 μM, Tocris, CAS no. 7421-40-1), and PPADS (100 μM, Tocris, CAS # 192575-19-2) were dissolved in the buffer used to fill the chambers. All substances were administered 10 to 15 min before the application of the corresponding stimulus, i.e., succinate or ACh (100 μM, Sigma-Aldrich, CAS no. 60-31-1), except GAP27, which was administered 45 min before the application of succinate. There was no washing step between applications of different substances. Maximum changes in *I*_SC_ within 2 min after application of substances were analyzed.

### Statistics

Statistical analyses were performed using Origin Pro (OriginLab Corporation, Northampton, MA, USA) or Prism 7 (GraphPad). For analysis of data collected in measurements of PTS, CBF, and *I*sc, first, the group with the largest sample size, i.e., responses to control stimuli such as ATP pooled from the various experiments, was tested for normality of distribution using the Kolmogorov-Smirnov test. All these cases revealed significant deviation from normal distribution, and, consequently, all data generated by these techniques were analyzed throughout by nonparametric tests, as specified in the respective figure legends. For analysis of data collected in Ca^2+^ imaging experiments, assumptions of normality and homogeneity of variance (Kolmogorov-Smirnov, Shapiro-Wilk) were tested before conducting the following statistical approaches. Student’s *t* test was used to measure the significance of the differences between two distributions. Multiple groups were compared using a one-way (multiple measurements) and two-way analysis of variance (ANOVA) with Tukey’s multiple comparisons test as a post hoc comparison. In case the results failed the test of normality, the Mann-Whitney or the Wilcoxon signed-rank test was used to measure the significance of the differences between two distributions, whereas multiple groups were compared using either the Kruskal-Wallis or the Friedman ANOVA with Dunn’s least test as a post hoc comparison. The probability of error level (alpha) was chosen to be 0.05. Unless otherwise stated, data are expressed as mean ± SD or median ± IQR (interquartile range: percentiles 25 to 75), depending on the normality of distribution. Inclusion/exclusion criteria were based on the viability of the preparations defined by response to control stimuli and are specified in the respective methods subchapter. If not otherwise stated, legends of the figures indicate the number of independent measurements. We used at least three mice per genotype, as required by the animal welfare committee.

## References

[1] M. I. Hollenhorst, R. Nandigama, S. B. Evers, I. Gamayun, N. Abdel Wadood, A. Salah, M. Pieper, A. Wyatt, A. Stukalov, A. Gebhardt, W. Nadolni, W. Burow, C. Herr, C. Beisswenger, S. Kusumakshi, F. Ectors, T. I. Kichko, L. Hübner, P. Reeh, A. Munder, S. M. Wienhold, M. Witzenrath, R. Bals, V. Flockerzi, U. Boehm, G. Krasteva-Christ, Bitter taste signaling in tracheal epithelial brush cells elicits innate immune responses to bacterial infection. J. Clin. Invest. 132, e150951 (2022). 35503420 10.1172/JCI150951 PMC9246383

[2] G. Krasteva, B. J. Canning, P. Hartmann, T. Z. Veres, T. Papadakis, C. Mühlfeld, K. Schliecker, Y. N. Tallini, A. Braun, H. Hackstein, N. Baal, E. Weihe, B. Schütz, M. Kotlikoff, I. Ibanez-Tallon, W. Kummer, Cholinergic chemosensory cells in the trachea regulate breathing. Proc. Natl. Acad. Sci. U.S.A. 108, 9478–9483 (2011). 21606356 10.1073/pnas.1019418108 PMC3111311

[3] C. J. Saunders, M. Christensen, T. E. Finger, M. Tizzano, Cholinergic neurotransmission links solitary chemosensory cells to nasal inflammation. Proc. Natl. Acad. Sci. U.S.A. 111, 6075–6080 (2014). 24711432 10.1073/pnas.1402251111 PMC4000837

[4] M. Tizzano, B. D. Gulbranson, A. Vandenbeuch, T. R. Clapp, J. P. Herman, H. M. Sibhatu, M. E. A. Churchhill, W. L. Silver, S. C. Kinnamon, T. E. Finger, Nasal chemosensory cells use bitter taste signaling to detect irritants and bacterial signals. Proc. Natl. Acad. Sci. U.S.A. 107, 3210–3215 (2010). 20133764 10.1073/pnas.0911934107 PMC2840287

[5] L. G. Bankova, D. F. Dwyer, E. Yoshimoto, S. Ualiyeva, J. W. McGinty, H. Raff, J. von Moltke, Y. Kanaoka, K. F. Austen, N. A. Barrett, The cysteinyl leukotriene 3 receptor regulates expansion of IL-25-producing airway brush cells leading to type 2 inflammation. Sci. Immunol. 3, eaat9453 (2018). 30291131 10.1126/sciimmunol.aat9453 PMC6599626

[6] R. J. Lee, J. M. Kofonow, P. L. Rosen, A. P. Siebert, B. Chen, L. Doghramji, G. Xiong, N. D. Adappa, J. N. Palmer, D. W. Kennedy, J. L. Kreindler, R. F. Margolskee, N. A. Cohen, Bitter and sweet taste receptors regulate human upper respiratory innate immunity. J. Clin. Invest. 124, 1393–1405 (2014). 24531552 10.1172/JCI72094 PMC3934184

[7] M. I. Hollenhorst, I. Jurastow, R. Nandigama, S. Appenzeller, L. Li, J. Vogel, S. Wiederhold, M. Althaus, M. Empting, J. Altmüller, A. K. H. Hirsch, V. Flockerzi, B. J. Canning, A. E. Saliba, G. Krasteva-Christ, Tracheal brush cells release acetylcholine in response to bitter tastants for paracrine and autocrine signaling. FASEB J. 34, 316–332 (2020). 31914675 10.1096/fj.201901314RR

[8] A. Perniss, S. Liu, B. Boonen, M. Keshavarz, A. L. Ruppert, T. Timm, U. Pfeil, A. Soultanova, S. Kusumakshi, L. Delventhal, Ö. Aydin, M. Pyrski, K. Deckmann, T. Hain, N. Schmidt, C. Ewers, A. Günther, G. Lochnit, V. Chubanov, T. Gudermann, J. Oberwinkler, J. Klein, K. Mikoshiba, T. Leinders-Zufall, S. Offermanns, B. Schütz, U. Boehm, F. Zufall, B. Bufe, W. Kummer, Chemosensory cell-derived acetylcholine drives tracheal mucociliary clearance in response to virulence-associated formyl peptides. Immunity 52, 683–699.e11 (2020). 32294408 10.1016/j.immuni.2020.03.005

[9] A. Wanner, M. Salathé, T. G. O’Riordan, Mucociliary clearance in the airways. Am. J. Respir. Crit. Care Med. 154, 1868–1902 (1996). 8970383 10.1164/ajrccm.154.6.8970383

[10] A. Hahn, J. Faulhaber, L. Srisawang, A. Stortz, J. J. Salomon, M. A. Mall, S. Frings, F. Möhrlen, Cellular distribution and function of ion channels involved in transport processes in rat tracheal epithelium. Physiol. Rep. 5, e13290 (2017). 28642338 10.14814/phy2.13290 PMC5492199

[11] K. Okuda, H. Dang, Y. Kobayashi, G. Carraro, S. Nakano, G. Chen, T. Kato, T. Asakura, R. C. Gilmore, L. C. Morton, R. E. Lee, T. Mascenik, W. N. Yin, S. M. Barbosa Cardenas, Y. K. O'Neal, C. E. Minnick, M. Chua, N. L. Quinney, M. Gentzsch, C. W. Anderson, A. Ghio, H. Matsui, T. Nagase, L. E. Ostrowski, B. R. Grubb, J. C. Olsen, S. H. Randell, B. R. Stripp, P. R. Tata, W. K. O'Neal, R. C. Boucher, Secretory cells dominate airway CFTR expression and function in human airway superficial epithelia. Am. J. Respir. Crit. Care Med. 203, 1275–1289 (2021). 33321047 10.1164/rccm.202008-3198OC PMC8456462

[12] R. J. Pack, L. H. Al-Ugaily, G. Morris, J. G. Widdicombe, The distribution and structure of cells in the tracheal epithelium of the mouse. Cell Tissue Res. 208, 65–84 (1980). 6248229 10.1007/BF00234174

[13] T. Paff, H. Omran, K. G. Nielsen, E. G. Haarman, Current and future treatments in primary ciliary dyskinesia. Int. J. Mol. Sci. 22, 9834 (2021). 34575997 10.3390/ijms22189834 PMC8470068

[14] M. Shteinberg, I. J. Haq, D. Polineni, J. C. Davies, Cystic fibrosis. Lancet 397, 2195–2211 (2021). 34090606 10.1016/S0140-6736(20)32542-3

[15] C. A. Pérez, L. Huang, M. Rong, J. A. Kozak, A. K. Preuss, H. Zhang, M. Max, R. F. Margolskee, A transient receptor potential channel expressed in taste receptor cells. Nat. Neurosci. 5, 1169–1176 (2002). 12368808 10.1038/nn952

[16] M. S. Nadjsombati, J. W. McGinty, M. R. Lyons-Cohen, J. B. Jaffe, L. DiPeso, C. Schneider, C. N. Miller, J. L. Pollack, G. A. Nagana Gowda, M. F. Fontana, D. J. Erle, M. S. Anderson, R. M. Locksley, D. Raftery, J. von Moltke, Detection of succinate by intestinal tuft cells triggers a type 2 innate immune circuit. Immunity 49, 33–41.e7 (2018). 30021144 10.1016/j.immuni.2018.06.016 PMC6084797

[17] C. Schneider, C. Schneider, C. E. O'Leary, J. von Moltke, H. E. Liang, Q. Y. Ang, P. J. Turnbaugh, S. Radhakrishnan, M. Pellizzon, A. Ma, R. M. Locksley, A metabolite-triggered tuft cell-ILC2 circuit drives small intestinal remodeling. Cell 174, 271–284.e14 (2018). 29887373 10.1016/j.cell.2018.05.014 PMC6046262

[18] M. Keshavarz, T. S. Faraj, A. L. Ruppert, U. Pfeil, Y. Schreiber, J. Klein, I. Brandenburger, G. Lochnit, S. Bhushan, A. Perniss, K. Deckmann, P. Hartmann, M. Meiners, P. Mermer, A. Rafiq, S. Winterberg, T. Papadakis, D. Thomas, C. Angioni, J. Oberwinkler, V. Chubanov, T. Gudermann, U. Gärtner, S. Offermanns, B. Schütz, W. Kummer, Cysteinyl leukotrienes and acetylcholine are biliary tuft cell cotransmitters. Sci. Immunol. 7, eabf6734 (2022). 35245090 10.1126/sciimmunol.abf6734

[19] S. A. Riquelme, C. Lozano, A. M. Moustafa, K. Liimatta, K. L. Tomlinson, C. Britto, S. Khanal, S. K. Gill, A. Narechania, J. M. Azcona-Gutiérrez, E. DiMango, Y. Saénz, P. Planet, A. Prince, CFTR-PTEN–dependent mitochondrial metabolic dysfunction promotes Pseudomonas aeruginosa airway infection. Sci. Transl. Med. 11, eaav4634 (2019). 31270271 10.1126/scitranslmed.aav4634 PMC6784538

[20] M. Tizzano, M. Cristofoletti, A. Sbarbati, T. E. Finger, Expression of taste receptors in solitary chemosensory cells of rodent airways. BMC Pulm. Med. 11, 3 (2011). 21232137 10.1186/1471-2466-11-3 PMC3031280

[21] D. T. Montoro, A. L. Haber, M. Biton, V. Vinarsky, B. Lin, S. E. Birket, F. Yuan, S. Chen, H. M. Leung, J. Villoria, N. Rogel, G. Burgin, A. M. Tsankov, A. Waghray, M. Slyper, J. Waldman, L. Nguyen, D. Dionne, O. Rozenblatt-Rosen, P. R. Tata, H. Mou, M. Shivaraju, H. Bihler, M. Mense, G. J. Tearney, S. M. Rowe, J. F. Engelhardt, A. Regev, J. Rajagopal, A revised airway epithelial hierarchy includes CFTR-expressing ionocytes. Nature 560, 319–324 (2018). 30069044 10.1038/s41586-018-0393-7 PMC6295155

[22] J. Yamashita, M. Ohmoto, T. Yamaguchi, I. Matsumoto, J. Hirota, Skn-1a/Pou2f3 functions as a master regulator to generate Trpm5-expressing chemosensory cells in mice. PLOS ONE 12, e0189340 (2017). 29216297 10.1371/journal.pone.0189340 PMC5720759

[23] J. T. Lock, I. Parker, I. F. Smith, A comparison of fluorescent Ca^2+^ indicators for imaging local Ca^2+^ signals in cultured cells. Cell Calcium 58, 638–648 (2015). 26572560 10.1016/j.ceca.2015.10.003 PMC4658286

[24] M. Tada, A. Takeuchi, M. Hashizume, K. Kitamura, M. Kano, A highly sensitive fluorescent indicator dye for calcium imaging of neural activity in vitro and in vivo. Eur. J. Neurosci. 39, 1720–1728 (2014). 24405482 10.1111/ejn.12476 PMC4232931

[25] L. F. Jaffe, Fast calcium waves. Cell calcium 48, 102–113 (2010). 20883893 10.1016/j.ceca.2010.08.007

[26] T. R. Clapp, K. F. Medler, S. Damak, R. F. Margolskee, S. C. Kinnamon, Mouse taste cells with G protein-coupled taste receptors lack voltage-gated calcium channels and SNAP-25. BMC Biol. 4, 7 (2006). 16573824 10.1186/1741-7007-4-7 PMC1444931

[27] S. Damak, M. Rong, K. Yasumatsu, Z. Kokrashvili, C. A. Pérez, N. Shigemura, R. Yoshida, B. Mosinger Jr, J. I. Glendinning, Y. Ninomiya, R. F. Margolskee, Trpm5 null mice respond to bitter, sweet, and umami compounds. Chem. Senses 31, 253–264 (2006). 16436689 10.1093/chemse/bjj027

[28] Z. Fu, T. Ogura, W. Luo, W. Lin, ATP and odor mixture activate TRPM5-expressing microvillous cells and potentially induce acetylcholine release to enhance supporting cell endocytosis in mouse main olfactory epithelium. Front. Cell. Neurosci. 12, 71 (2018). 29615870 10.3389/fncel.2018.00071 PMC5869921

[29] T. R. Clapp, L. M. Stone, R. F. Margolskee, S. C. Kinnamon, Immunocytochemical evidence for co-expression of Type III IP3 receptor with signaling components of bitter taste transduction. BMC Neurosci. 2, 6 (2001). 11346454 10.1186/1471-2202-2-6 PMC31433

[30] W. Lei, W. Ren, M. Ohmoto, J. F. Urban, I. Matsumoto, R. F. Margolskee, P. Jiang, Activation of intestinal tuft cell-expressed Sucnr1 triggers type 2 immunity in the mouse small intestine. Proc. Natl. Acad. Sci. U.S.A. 115, 5552–5557 (2018). 29735652 10.1073/pnas.1720758115 PMC6003470

[31] P. Chamero, J. Weiss, M. T. Alonso, M. Rodríguez-Prados, C. Hisatsune, K. Mikoshiba, T. Leinders-Zufall, F. Zufall, Type 3 inositol 1,4,5-trisphosphate receptor is dispensable for sensory activation of the mammalian vomeronasal organ. Sci. Rep. 7, 10260 (2017). 28860523 10.1038/s41598-017-09638-8 PMC5579292

[32] F. J. Martin, A. S. Prince, TLR2 regulates gap junction intercellular communication in airway cells. J. Immunol. 180, 4986–4993 (2008). 18354224 10.4049/jimmunol.180.7.4986 PMC2712445

[33] K. Droguett, M. Rios, D. V. Carreno, C. Navarrete, C. Fuentes, M. Villalon, N. P. Barrera, An autocrine ATP release mechanism regulates basal ciliary activity in airway epithelium. J. Physiol. 595, 4755–4767 (2017). 28422293 10.1113/JP273996 PMC5509870

[34] L. Homolya, T. H. Steinberg, R. C. Boucher, Cell to cell communication in response to mechanical stress via bilateral release of ATP and UTP in polarized epithelia. J. Cell Biol. 150, 1349–1360 (2000). 10995440 10.1083/jcb.150.6.1349 PMC2150709

[35] L. W. Plasschaert, R. Žilionis, R. Choo-Wing, V. Savova, J. Knehr, G. Roma, A. M. Klein, A. B. Jaffe, A single-cell atlas of the airway epithelium reveals the CFTR-rich pulmonary ionocyte. Nature 560, 377–381 (2018). 30069046 10.1038/s41586-018-0394-6 PMC6108322

[36] S. Ualiyeva, N. Hallen, Y. Kanaoka, C. Ledderose, I. Matsumoto, W. G. Junger, N. A. Berrett, L. G. Bankova, Airway brush cells generate cysteinyl leukotrienes through the ATP sensor P2Y2. Sci. Immunol. 5, eaax7224 (2020). 31953256 10.1126/sciimmunol.aax7224 PMC7176051

[37] S. Boitano, W. H. Evans, Connexin mimetic peptides reversibly inhibit Ca^2+^ signaling through gap junctions in airway cells. Am. J. Physiol. Lung Cell. Mol. Physiol. 279, L623–L630 (2000). 11000121 10.1152/ajplung.2000.279.4.L623

[38] M. L. Cotter, S. Boitano, J. Vagner, J. M. Burt, Lipidated connexin mimetic peptides potently inhibit gap junction-mediated Ca^2+^−wave propagation. Am. J. Physiol. Cell Physiol. 315, C141–C154 (2018). 29631365 10.1152/ajpcell.00156.2017 PMC6139506

[39] L. K. Choo, F. J. Mitchelson, Comparison of the affinity constant of some muscarinic receptor antagonists with their displacement of [3H]quinuclidinyl benzilate binding in atrial and ileal longitudinal muscle of the guinea-pig. J. Pharm. Pharmacol. 37, 656–658 (1985). 2867189 10.1111/j.2042-7158.1985.tb05106.x

[40] V. Abudara, J. Bechberger, M. Freitas-Andrade, M. De Bock, N. Wang, G. Bultynck, C. C. Naus, L. Leybaert, C. Giaume, The connexin43 mimetic peptide Gap19 inhibits hemichannels without altering gap junctional communication in astrocytes. Front. Cell. Neurosci. 8, 306 (2014). 25374505 10.3389/fncel.2014.00306 PMC4204617

[41] A. L. Chang-Graham, A. L. Chang-Graham, J. L. Perry, M. A. Engevik, K. A. Engevik, F. J. Scribano, J. T. Gebert, H. A. Danhof, J. C. Nelson, J. S. Kellen, A. C. Strtak, N. P. Sastri, M. K. Estes, R. A. Britton, J. Versalovic, J. M. Hyser, Rotavirus induces intercellular calcium waves through ADP signaling. Science 370, eabc3621 (2020). 33214249 10.1126/science.abc3621 PMC7957961

[42] M. K. Klein, R. V. Haberberger, P. Hartmann, P. Faulhammer, K. S. Lips, B. Krain, J. Wess, W. Kummer, P. König, Muscarinic receptor subtypes in cilia-driven transport and airway epithelial development. Eur. Respir. J. 33, 1113–1121 (2009). 19213795 10.1183/09031936.00015108 PMC3895332

[43] D. M. Morse, j. L. Smullen, C. W. Davis, Differential effects of UTP, ATP, and adenosine on ciliary activity of human nasal epithelial cells. Am. J. Physiol. Cell Physiol. 280, 1485–1497 (2001). 10.1152/ajpcell.2001.280.6.C1485 11350744

[44] S. L. Winters, C. W. Davis, R. C. Boucher, Mechanosensitivity of mouse tracheal ciliary beat frequency: Roles for Ca^2+^, purinergic signaling, tonicity, and viscosity. Am. J. Physiol. Cell Physiol. 292, L614–L624 (2007). 10.1152/ajplung.00288.2005 16963528

[45] M. I. Hollenhorst, K. S. Lips, M. Wolff, J. Wess, S. Gerbig, Z. Takats, W. Kummer, M. Fronius, Luminal cholinergic signalling in airway lining fluid: A novel mechanism for activating chloride secretion via Ca^2+^-dependent Cl^−^ and K^+^ channels. Br. J. Pharmacol. 166, 1388–1402 (2012). 22300281 10.1111/j.1476-5381.2012.01883.x PMC3417454

[46] M. I. Hollenhorst, K. S. Lips, W. Kummer, M. Fronius, Nicotine-induced activation of soluble adenylyl cyclase participates in ion transport regulation in mouse tracheal epithelium. Life Sci. 91, 1009–1012 (2012). 22771693 10.1016/j.lfs.2012.06.027

[47] M. I. Hollenhorst, K. Richter, M. Fronius, Ion transport by pulmonary epithelia. J. Biomed. Biotechnol. 2011, 174306 (2011). 22131798 10.1155/2011/174306 PMC3205707

[48] J. D. McCann, M. J. Welsh, Regulation of Cl- and K+ channels in airway epithelium. Annu. Rev. Physiol. 52, 115–135 (1990). 2158763 10.1146/annurev.ph.52.030190.000555

[49] A. Gianotti, L. Ferrera, A. R. Philp, E. Caci, O. Zegarra-Moran, L. J. Galietta, C. A. Flores, Pharmacological analysis of epithelial chloride secretion mechanisms in adult murine airways. Eur. J. Pharmacol. 781, 100–108 (2016). 27063443 10.1016/j.ejphar.2016.04.007

[50] A. Hahn, J. J. Salomon, D. Leitz, D. Feigenbutz, L. Korsch, I. Lisewski, K. Schrimpf, P. Millar-Büchner, M. A. Mall, S. Frings, F. Möhrlen, Expression and function of Anoctamin 1/TMEM16A calcium-activated chloride channels in airways of in vivo mouse models for cystic fibrosis research. Pflug. Arch. Eur. 470, 1335–1348 (2018). 10.1007/s00424-018-2160-x 29860639

[51] J. Lin, Y. Jiang, Y. Liu, H. Tang, D. Jiang, TMEM16A mediates the hypersecretion of mucus induced by Interleukin-13. Exp. Cell Res. 334, 260–269 (2015). 25770012 10.1016/j.yexcr.2015.02.026

[52] J. R. Rock, W. K. O’Neal, S. E. Gabriel, S. H. Randell, B. D. Harfe, R. C. Boucher, B. R. Grubb, Transmembrane protein 16A (TMEM16A) is a Ca^2+^-regulated Cl^−^ secretory channel in mouse airways. J. Biol. Chem. 284, 14875–14880 (2009). 19363029 10.1074/jbc.C109.000869 PMC2685669

[53] A. Townsend-Nicholson, B. F. King, S. S. Wildman, G. Burnstock, Molecular cloning, functional characterization and possible cooperativity between the murine P2X4 and P2X4a receptors. Brain Res. Mol. Brain Res. 64, 246–254 (1999). 9931497 10.1016/s0169-328x(98)00328-3

[54] S. Esposito, V. R. Villella, F. Rossin, A. Tosco, V. Raia, A. Luciani, Succinate links mitochondria to deadly bacteria in cystic fibrosis. Ann. Transl. Med. 7(Suppl. 8), S263 (2019). 32015982 10.21037/atm.2019.12.49 PMC6976446

[55] S. A. Riquelme, A. Prince, Airway immunometabolites fuel Pseudomonas aeruginosa infection. Respir. Res. 21, 326 (2020). 33302964 10.1186/s12931-020-01591-x PMC7731785

[56] A. Banerjee, C. A. Herring, B. Chen, H. Kim, A. J. Simmons, A. N. Southard-Smith, M. M. Allaman, J. R. White, M. C. Macedonia, E. T. Mckinley, M. A. Ramirez-Solano, E. A. Scoville, Q. Liu, K. T. Wilson, R. J. Coffey, M. K. Washington, J. A. Goettel, K. S. Lau, Succinate produced by intestinal microbes promotes specification of tuft cells to suppress ileal inflammation. Gastroenterology 159, 2101–2115.e5 (2020). 32828819 10.1053/j.gastro.2020.08.029 PMC7725941

[57] K. R. Clarke, The migration route of the third stage larvae of Nippostrongylus brasiliensis (Travassos, 1914). J. Helminthol. 41, 285–290 (1967). 6057041 10.1017/s0022149x00021817

[58] J. J. Reece, M. C. Siracusa, A. L. Scott, Innate immune responses to lung-stage helminth infection induce alternatively activated alveolar macrophages. Infect. Immun. 74, 4970–4981 (2006). 16926388 10.1128/IAI.00687-06 PMC1594865

[59] L. B. Roberts, C. Schnoeller, R. Berkachy, M. Darby, J. Pillaye, M. J. Oudhoff, N. Parmar, C. Mackowiak, D. Sedda, V. Quesniaux, B. Ryffel, R. Vaux, K. Gounaris, S. Berrard, D. R. Withers, W. G. C. Horsnell, M. E. Selkirk, Acetylcholine production by group 2 innate lymphoid cells promotes mucosal immunity to helminths. Sci. Immunol. 6, eabd0359 (2021). 33674321 10.1126/sciimmunol.abd0359

[60] S. P. Singh, J. C. Katiyar, V. M. Srivastava, Enzymes of the tricarboxylic acid cycle in *Ancylostoma ceylanicum* and *Nippostrongylus brasiliensis*. J. Parasitol. Res. 78, 24–29 (1992). 1738065

[61] M. I. Hollenhorst, P. Kumar, M. Zimmer, A. Salah, S. Maxeiner, M. I. Elhawy, S. B. Evers, V. Flockerzi, T. Gudermann, V. Chubanov, U. Boehm, G. Krasteva-Christ, Taste receptor activation in tracheal brush cells by denatonium modulates ENaC channels via Ca^2+^, cAMP and ACh. Cell 11, 2411 (2022). 10.3390/cells11152411 PMC9367940 35954259

[62] C. Lasconi, S. Pifferi, A. Hernandez-Clavijo, F. Merigo, M. P. Cecchini, K. Y. Gonzalez-Velandia, E. Agostinelli, A. Sbarbati, A. Menini, Bitter tastants and artificial sweeteners activate a subset of epithelial cells in acute tissue slices of the rat trachea. Sci. Rep. 9, 8834 (2019). 31222082 10.1038/s41598-019-45456-w PMC6586933

[63] E. A. Sell, L. H. Tan, J. V. Bosso, J. N. Palmer, N. D. Adappa, R. J. Lee, M. A. Kohanski, D. R. Reed, N. A. Cohen, Microbial metabolite succinate activates solitary chemosensory cells in the human sinonasal epithelium. Int. Forum Allergy Rhinol. 2023, 1–10 (2023). 10.1002/alr.23104 36565436

[64] W. Yu, T. O. Moninger, M. V. Rector, D. A. Stoltz, M. J. Welsh, Pulmonary neuroendocrine cells sense succinate to stimulate myoepithelial cell contraction. Dev. Cell 57, 2221–2236.e5 (2022). 36108628 10.1016/j.devcel.2022.08.010 PMC9762774

[65] M. E. Kotas, C. M. Moore, J. G. Gurrola, S. D. Pletcher, A. N. Goldberg, R. Alvarez, S. Yamato, P. E. Bratcher, C. A. Shaughnessy, P. L. Zeitlin, I. H. Zhang, Y. Li, M. T. Montgomery, K. Lee, E. K. Cope, R. M. Locksley, M. A. Seibold, E. D. Gordon, IL-13-programmed airway tuft cells produce PGE2, which promotes CFTR-dependent mucociliary function. JCI Insight. 7, e159832 (2022). 35608904 10.1172/jci.insight.159832 PMC9310525

[66] S. Kusumakshi, A. Voigt, S. Hübner, I. Hermans-Borgmeyer, A. Ortalli, M. Pyrski, J. Dörr, F. Zufall, V. Flockerzi, W. Meyerhof, J. P. Montmayeur, U. Boehm, A binary genetic approach to characterize TRPM5 cells in mice. Chem. Senses 40, 413–425 (2015). 25940069 10.1093/chemse/bjv023

[67] L. Madisen, A. R. Garner, D. Shimaoka, A. S. Chuong, N. C. Klapoetke, L. Li, A. van der Bourg, Y. Niino, L. Egolf, C. Monetti, H. Gu, M. Mills, A. Cheng, B. Tasic, T. N. Nguyen, S. M. Sunkin, A. Benucci, A. Nagy, A. Miyawaki, F. Helmchen, R. M. Empson, T. Knöpfel, E. S. Boyden, R. C. Reid, M. Carandini, H. Zeng, Transgenic mice for intersectional targeting of neural sensors and effectors with high specificity and performance. Neuron 85, 942–958 (2015). 25741722 10.1016/j.neuron.2015.02.022 PMC4365051

[68] L. Madisen, T. A. Zwingman, S. M. Sunkin, S. W. Oh, H. A. Zariwala, H. Gu, L. L. Ng, R. D. Palmiter, M. J. Hawrylycz, A. R. Jones, E. S. Lein, H. Zeng, A robust and high-throughput Cre reporting and characterization system for the whole mouse brain. Nat. Neurosci. 13, 133–140 (2010). 20023653 10.1038/nn.2467 PMC2840225

[69] H. Jiang, Y. Kuang, Y. Wu, W. Xie, M. I. Simon, D. Wu, Roles of phospholipase C β2 in chemoattractant-elicited responses. Proc. Natl. Acad. Sci. U.S.A. 94, 7971–7975 (1997). 9223297 10.1073/pnas.94.15.7971 PMC21539

[70] W. Lin, E. A. D. Ezekwe, Z. Zhao, E. R. Liman, D. Restrepo, TRPM5-expressing microvillous cells in the main olfactory epithelium. BMC Neurosci. 9, 114 (2008). 19025635 10.1186/1471-2202-9-114 PMC2648980

[71] M. Pyrski, E. Eckstein, A. Schmidt, B. Bufe, J. Weiss, V. Chubanov, U. Boehm, F. Zufall, Trpm5 expression in the olfactory epithelium. Mol. Cell. Neurosci. 80, 75–88 (2017). 28188885 10.1016/j.mcn.2017.02.002

[72] I. Matsumoto, M. Ohmoto, M. Narukawa, Y. Yoshihara, K. Abe, Skn-1a (Pou2f3) specifies taste receptor cell lineage. Nat. Neurosci. 14, 685–687 (2011). 21572433 10.1038/nn.2820 PMC3390744

[73] S. Zurborg, A. Piszczek, C. Martínez, P. Hublitz, M. Al Banchaabouchi, P. Moreira, E. Perlas, P. A. Heppenstall, Generation and characterization of an advillin-Cre driver mouse line. Mol. Pain 7, 1744-8069-7-66 (2011). 10.1186/1744-8069-7-66 PMC3185264 21906401

[74] S. Ualiyeva, E. Yoshimoto, N. A. Barrett, L. G. Bankova, Isolation and quantitative evaluation of brush cells from mouse tracheas. J. Vis. Exp. 12, 10.3791/59496, (2019). PMC6830433 31259891

[75] S. L. Wolock, R. Lopez, A. M. Klein, Scrublet: Computational identification of cell doublets in single-cell transcriptomic data. Cell Syst. 8, 281–291.e9 (2019). 30954476 10.1016/j.cels.2018.11.005 PMC6625319

[76] R. Satija, J. A. Farrell, D. Gennert, A. F. Schier, A. Regev, Spatial reconstruction of single-cell gene expression data. Nat. Biotech. 33, 495–502 (2015). 10.1038/nbt.3192 PMC4430369 25867923

[77] A.-C. Trouillet, M. Keller, J. Weiss, T. Leinders-Zufall, L. Birnbaumer, F. Zufall, P. Chamero, Central role of G protein Gαi2 and Gαi2+ vomeronasal neurons in balancing territorial and infant-directed aggression of male mice. Proc. Natl. Acad. Sci. U.S.A. 116, 5135–5143 (2019). 30804203 10.1073/pnas.1821492116 PMC6421405

[78] T. Leinders-Zufall, T. Ishii, P. Chamero, P. Hendrix, L. Oboti, A. Schmid, S. Kircher, M. Pyrski, S. Akiyoshi, M. Khan, E. Vaes, F. Zufall, P. Mombaerts, A family of nonclassical class I MHC genes contributes to ultrasensitive chemodetection by mouse vomeronasal sensory neurons. J. Neurosci. 34, 5121–5133 (2014). 24719092 10.1523/JNEUROSCI.0186-14.2014 PMC4050176

[79] T. Leinders-Zufall, T. Ishii, P. Mombaerts, F. Zufall, T. Boehm, Structural requirements for the activation of vomeronasal sensory neurons by MHC peptides. Nat. Neurosci. 12, 1551–1558 (2009). 19935653 10.1038/nn.2452

[80] P. Chamero, V. Katsoulidou, P. Hendrix, B. Bufe, R. Roberts, H. Matsunami, J. Abramowitz, L. Birnbaumer, F. Zufall, T. Leinders-Zufall, G protein G(alpha)o is essential for vomeronasal function and aggressive behavior in mice. Proc. Natl. Acad. Sci. U.S.A. 108, 12898–12903 (2011). 21768373 10.1073/pnas.1107770108 PMC3150917

[81] T. Leinders-Zufall, P. Brennan, P. Widmayer, A. Maul-Pavicic, M. Jäger, X. H. Li, H. Breer, F. Zufall, T. Boehm, MHC class I peptides as chemosensory signals in the vomeronasal organ. Science 306, 1033–1037 (2004). 15528444 10.1126/science.1102818

[82] A. Perniss, A. Latz, I. Boseva, T. Papadakis, C. Dames, C. Meisel, A. Meisel, P. Scholze, W. Kummer, G. Krasteva-Christ, Acute nicotine administration stimulates ciliary activity via α3β4 nAChR in the mouse trachea. Int. Immunopharmacol. 84, 106496 (2020). 32304995 10.1016/j.intimp.2020.106496

[83] A. Perniss, N. Schmidt, C. Gurtner, K. Dietert, O. Schwengers, M. Weigel, J. Hempe, C, Ewers, U. Pfeil, U. Gärtner, A. D. Gruber, T. Hain, W. Kummer, *Bordetella pseudohinzii* targets cilia and impairs tracheal cilia-driven transport in naturally acquired infection in mice. Sci. Rep. 8, 5681 (2018). 29632402 10.1038/s41598-018-23830-4 PMC5890243

[84] S. Tunaru, R. Bonnavion, I. Brandenburger, J. Preussner, D. Thomas, K. Scholich, S. Offermanns, 20-HETE promotes glucose-stimulated insulin secretion in an autocrine manner through FFAR1. Nat. Commun. 9, 177 (2018). 29330456 10.1038/s41467-017-02539-4 PMC5766607

[85] V. Baubet, H. L. Mouellic, A. K. Campbell, E. Lucas-Meunier, P. Fossier, P. Brulet, Chimeric green fluorescent protein-aequorin as bioluminescent Ca^2+^ reporters at the single-cell level. Proc. Natl. Acad. Sci. U.S.A. 97, 7260–7265 (2000). 10860991 10.1073/pnas.97.13.7260 PMC16533

