# Supplementary material for: A succinate/SUCNR1-brush cell defense program in the tracheal epithelium
Source: Sci Adv. 2023 Aug 2;9(31):eadg8842. doi: 10.1126/sciadv.adg8842 (PMC10396310; doi:10.1126/sciadv.adg8842)
Supplement: Supplementary file 1 — Figs. S1 to S13 Table S1 Legends for movies S1 and S2 [file sciadv.adg8842_sm.pdf]

Science Advances
AAAS

Supplementary Materials for

A succinate/SUCNR1-brush cell defense program in the tracheal epithelium

Alexander Perniss *et al.*

Corresponding author:

Wolfgang Kummer, wolfgang.kummer@anatomie.med.uni-giessen.de; Frank Zufall, frank.zufall@uks.eu; Trese Leinders-Zufall, trese.leinders@uks.eu

Sci. Adv. **9**, eadg8842 (2023)
DOI: 10.1126/sciadv.adg8842

The PDF file includes:

Figs. S1 to S13
Table S1
Legends for movies S1 and S2

Other Supplementary Material for this manuscript includes the following:Mov

Movies S1 and S2

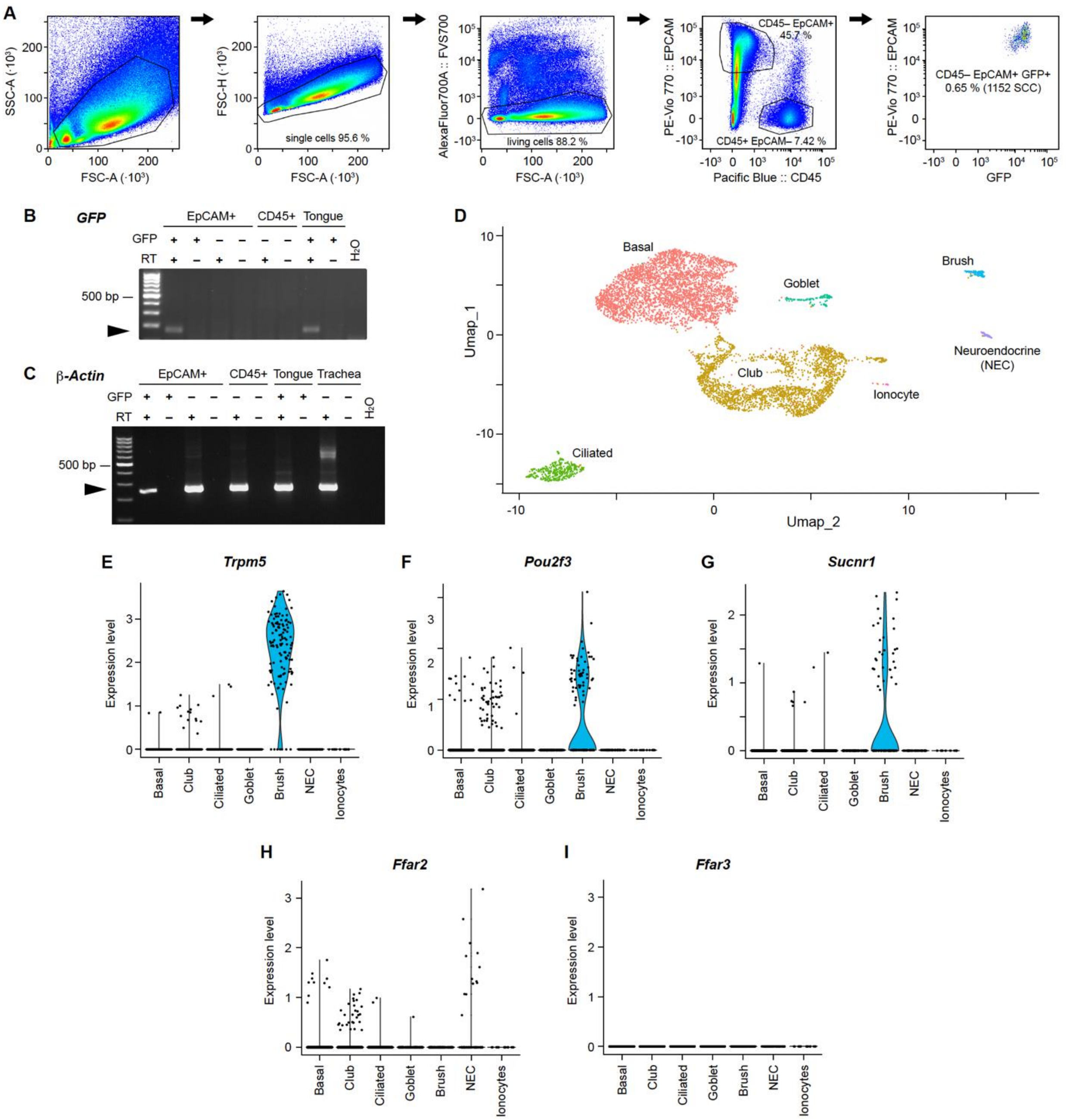
A
SSC-A (·10³)
FSC-A (·10³)
FSC-H (·10³)
single cells 95.6 %
AlexaFluor700A :: FVS700
living cells 88.2 %
PE-Vio 770 :: EPCAM
CD45– EpCAM+ 45.7 %
CD45+ EpCAM– 7.42 %
Pacific Blue :: CD45
CD45– EpCAM+ GFP+ 0.65 % (1152 SCC)
GFP
B
GFP
EpCAM+
CD45+
Tongue
RT
H2O
500 bp
C
β-Actin
EpCAM+
CD45+
Tongue
Trachea
GFP
RT
H2O
500 bp
D
Basal
Goblet
Brush
Neuroendocrine (NEC)
Club
Ionocyte
Ciliated
Umap_1
Umap_2
E
Trpm5
F
Pou2f3
G
Sucnr1
H
Ffar2
I
Ffar3
Expression level
Basal
Club
Ciliated
Goblet
Brush
NEC
Ionocytes

Figure S1

Fig. S1. *Sucnr1* is exclusively expressed by brush cells in the tracheal epithelium.
(A to C) Supporting material for RT-experiments of tracheal cells isolated by FACS shown in Fig. 1 A-B. (A) Gating strategies for brush cells, identified by GFP expression under the promotor of *Trpm5* and the presence of EpCAM, Epithelial cells identified by EpCAM expression and immune cells identified by CD45 expression. Tracheas of *Trpm5*-GFP mice were digested and cells were stained with antibodies against EpCAM and CD45. **(B and C)** RT-PCR of cells isolated by FACS with primers for GFP (B) and β-actin (C). RT ±, samples processed with and without reverse transcriptase; H_2O, sample without cDNA; tongue and whole trachea served as positive controls. **(D and E)** *In silico* analysis of published single-cell mRNA sequencing data (GSE103354) of murine tracheal epithelial cells (20). (D) SPRING plot (Uniform Manifold Approximation and Projection, UMAP) with seven distinct cell clusters, namely basal, goblet club, ciliated, brush cells, neuroendocrine cells (NEC), ionocytes. **(E to I)** Violin plots showing expression of *Trpm5* (E), *Pou2f3* (F), *Sucnr1* (G), *Ffar2* (H) and *Ffar3* (I) in distinct cell populations.

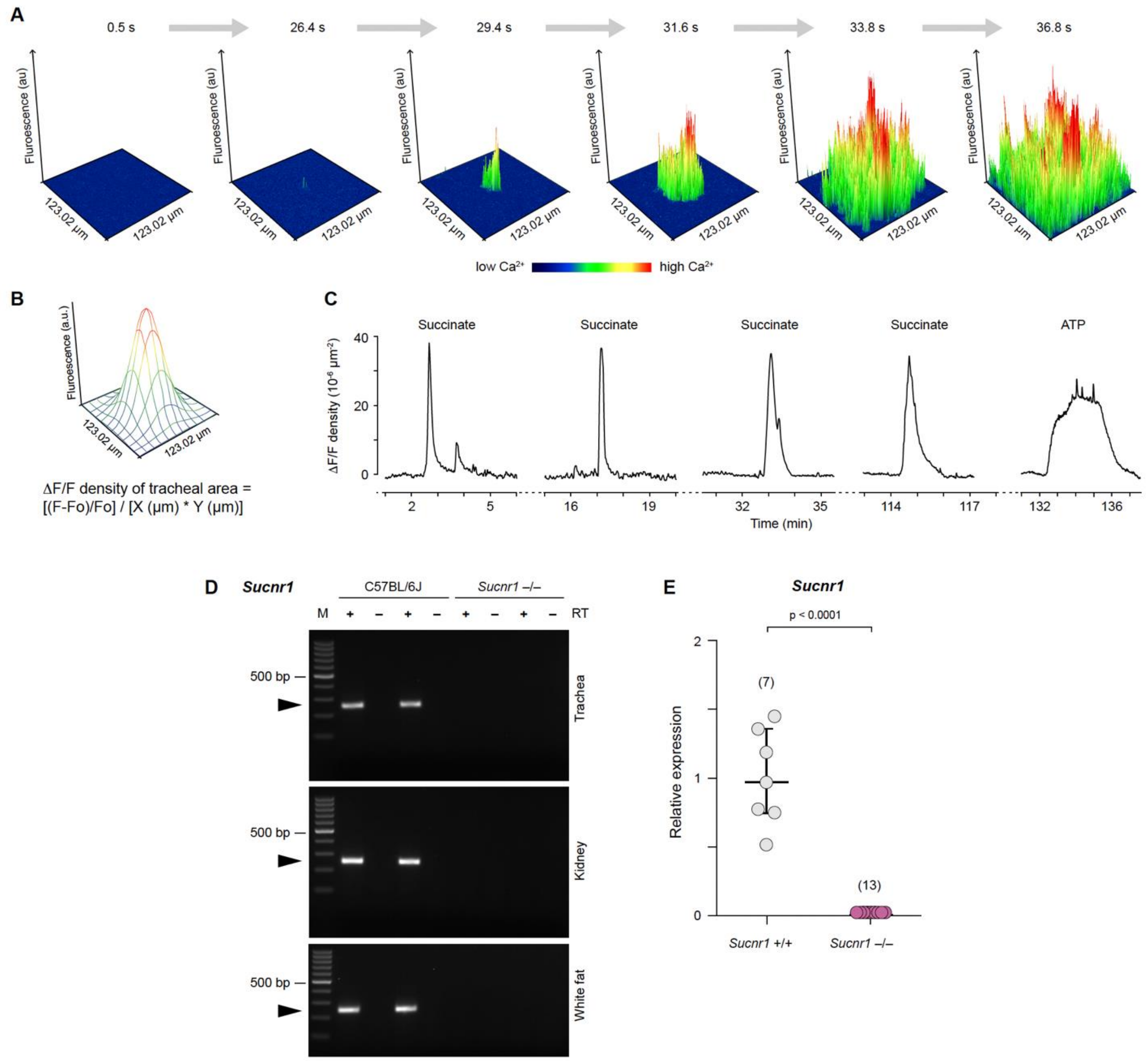

Fig. S2. Analyses of succinate-evoked tracheal Ca^{2+} waves. (A) 3D activity patterns (surface plots) of a tracheal succinate-evoked Ca^{2+} wave (1 mM) at different time points with a standard FOV of 15,134 μm^2. Same experiment and time frames as in Fig. 2D. **(B)** Calculation method of ΔF/F density values for succinate-evoked Ca^{2+} waves. **(C)** Succinate-evoked Ca^{2+} waves can be repeated multiple times in a given trachea without significant run-down. ATP (20 µM) served as a control stimulus at the end of the experiment. **(D)** RT-PCR of trachea, kidney, and white fat (abdominal) of C57BL/6J and *Sucnr1*$^{-/-}$ mice with primers for *Sucnr1*, agarose gel electrophoresis; RT ±, samples processed with and without reverse transcriptase. **(E)** Relative expression of *Sucnr1* in whole tracheas of *Sucnr1*$^{+/-}$ and *Sucnr1*$^{-/-}$ mice assessed by real-time RT-PCR. Data, median ± IQR, number of tracheas is given in parentheses; Mann-Whitney-test.

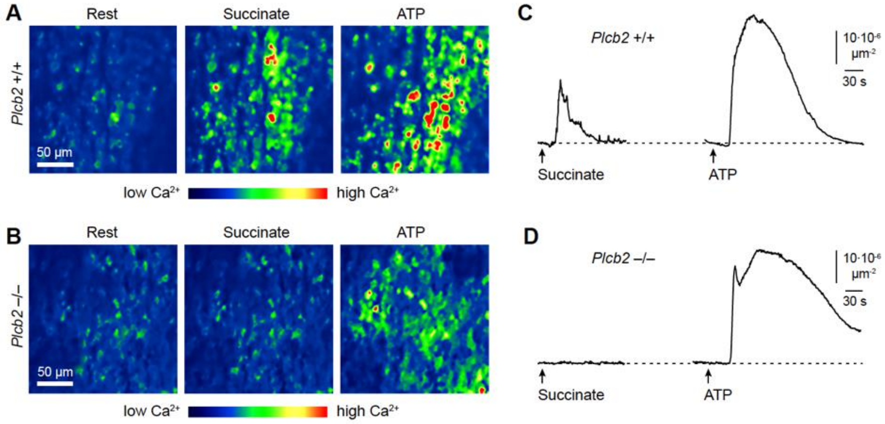

Fig. S3. Tracheal succinate-evoked Ca^{2+} waves but not ATP-evoked Ca^{2+} responses are eliminated in *Plcb2*-deficient mice. (**A to D**) Representative succinate-evoked Ca^{2+} waves (1 mM) and their time dependence from tracheas of *Trpm5*-tdTomato;*Plcb2*$^{–/–}$ mice and their wildtype littermate controls (*Trpm5*-tdTomato;*Plcb2*$^{+/+}$). ATP (20 µM) served as a control stimulus. Images **(A, B)** of the mean peak Ca^{2+} waves are mean fluorescence intensities (18 frames, 512 x 512 pixels, Δt = 0.74 s, time frame = 13.32 s) at rest and during ligand-evoked peak responses. Succinate 1 mM; ATP 20 µM.

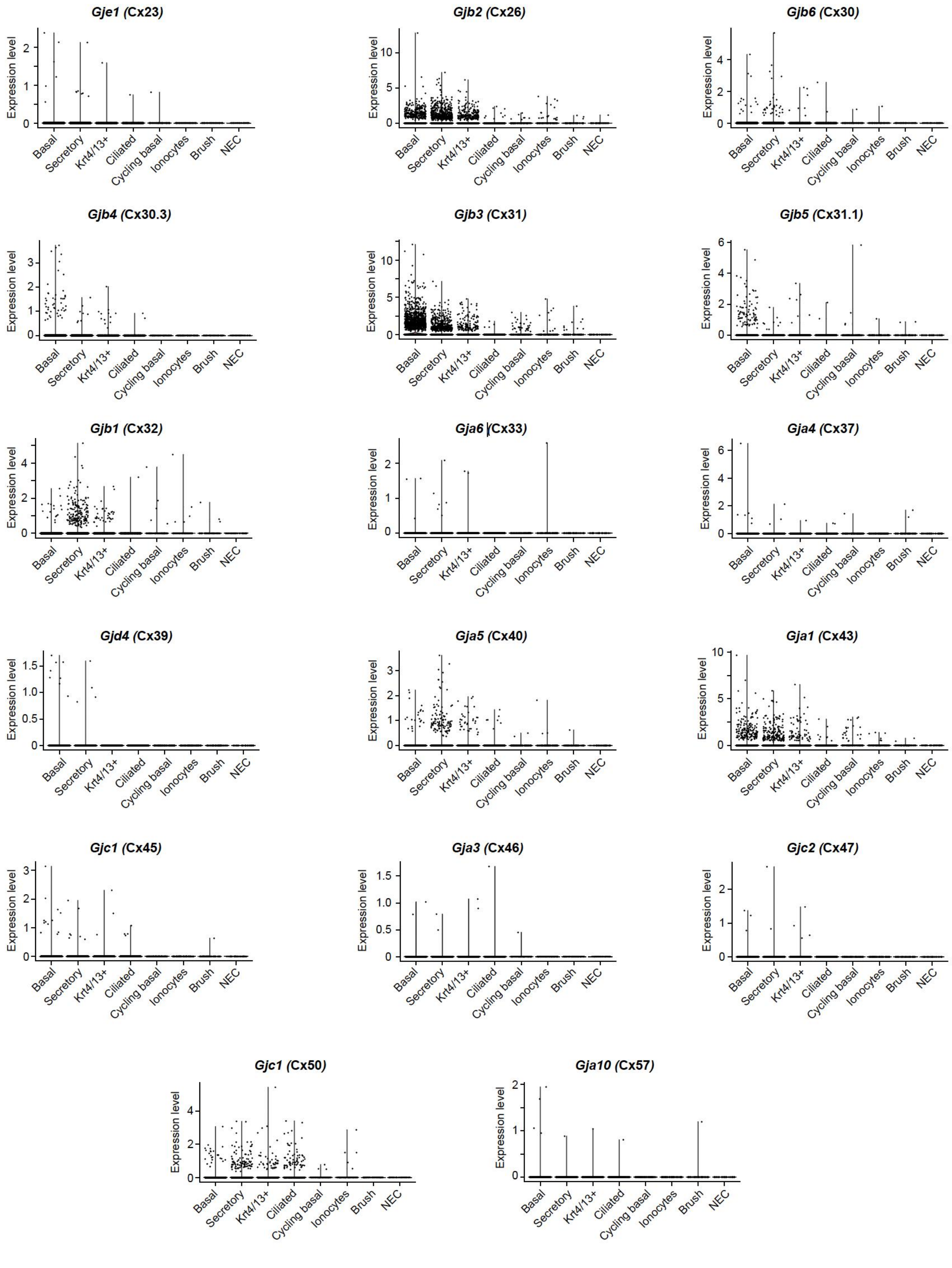
Gje1 (Cx23)
Gjb2 (Cx26)
Gjb6 (Cx30)
Gjb4 (Cx30.3)
Gjb3 (Cx31)
Gjb5 (Cx31.1)
Gjb1 (Cx32)
Gja6 (Cx33)
Gja4 (Cx37)
Gjd4 (Cx39)
Gja5 (Cx40)
Gja1 (Cx43)
Gjc1 (Cx45)
Gja3 (Cx46)
Gjc2 (Cx47)
Gjc1 (Cx50)
Gja10 (Cx57)
Expression level
Basal
Secretory
Krt4/13+
Ciliated
Cycling basal
Ionocytes
Brush
NEC

Figure S4

Fig. S4. Tracheal epithelial cells express gap junction proteins. *In silico*-analysis of single-cell RNA sequencing data of murine tracheal epithelial cells (data set GSE102580) (35). Violin plots show expression of connexins (Cx) in distinct cell clusters. Commonly used abbreviates for connexin proteins given in parentheses, corresponding gene names in italics.

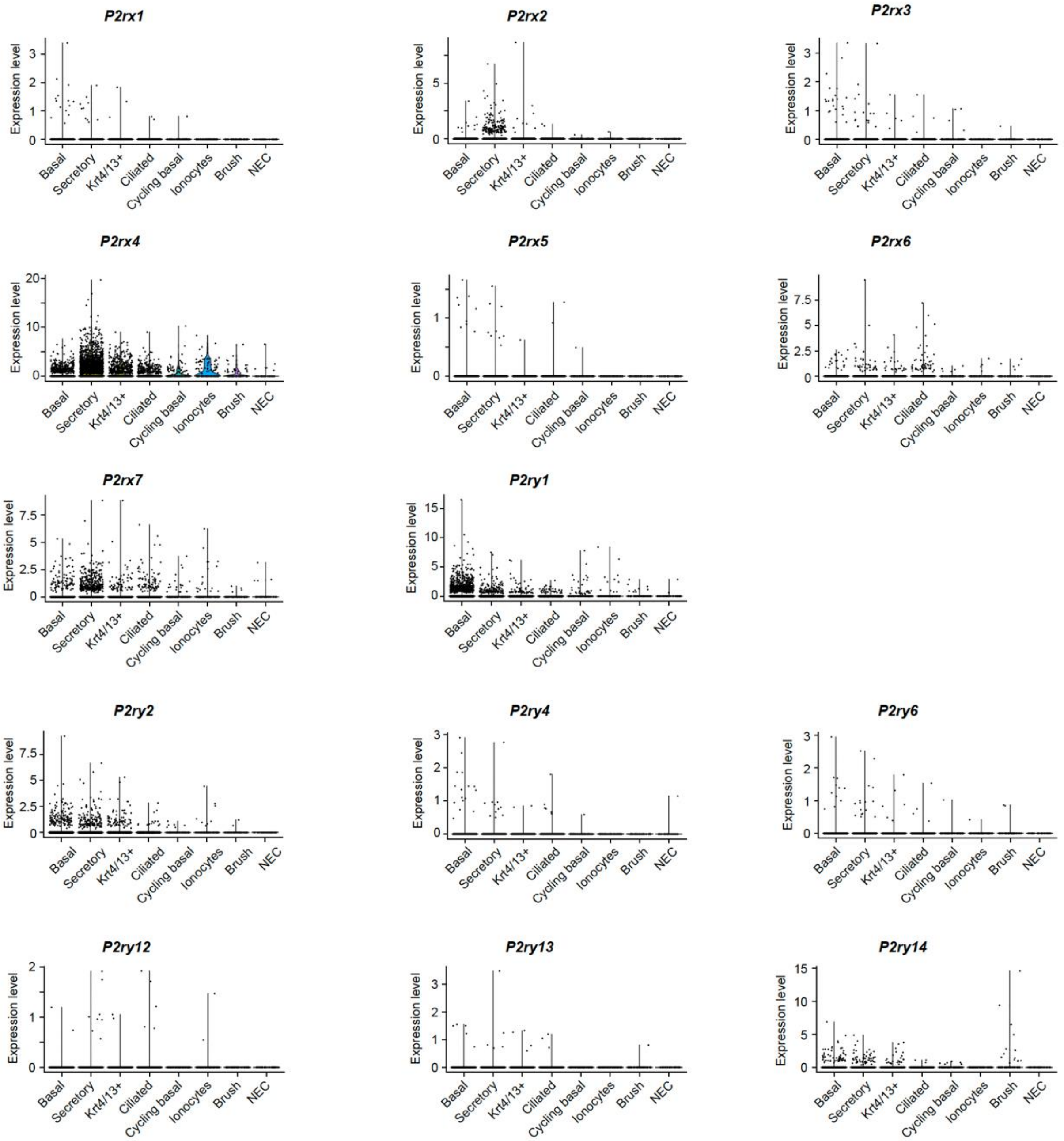

Fig. S5. Tracheal epithelial cells express purinergic P2 receptors. *In silico*-analysis of single-cell RNA sequencing data of murine tracheal epithelial cells (data set GSE102580) (35). Violin plots show expression of ionotropic P2X and metabotropic P2Y recetords in distinct cell clusters.

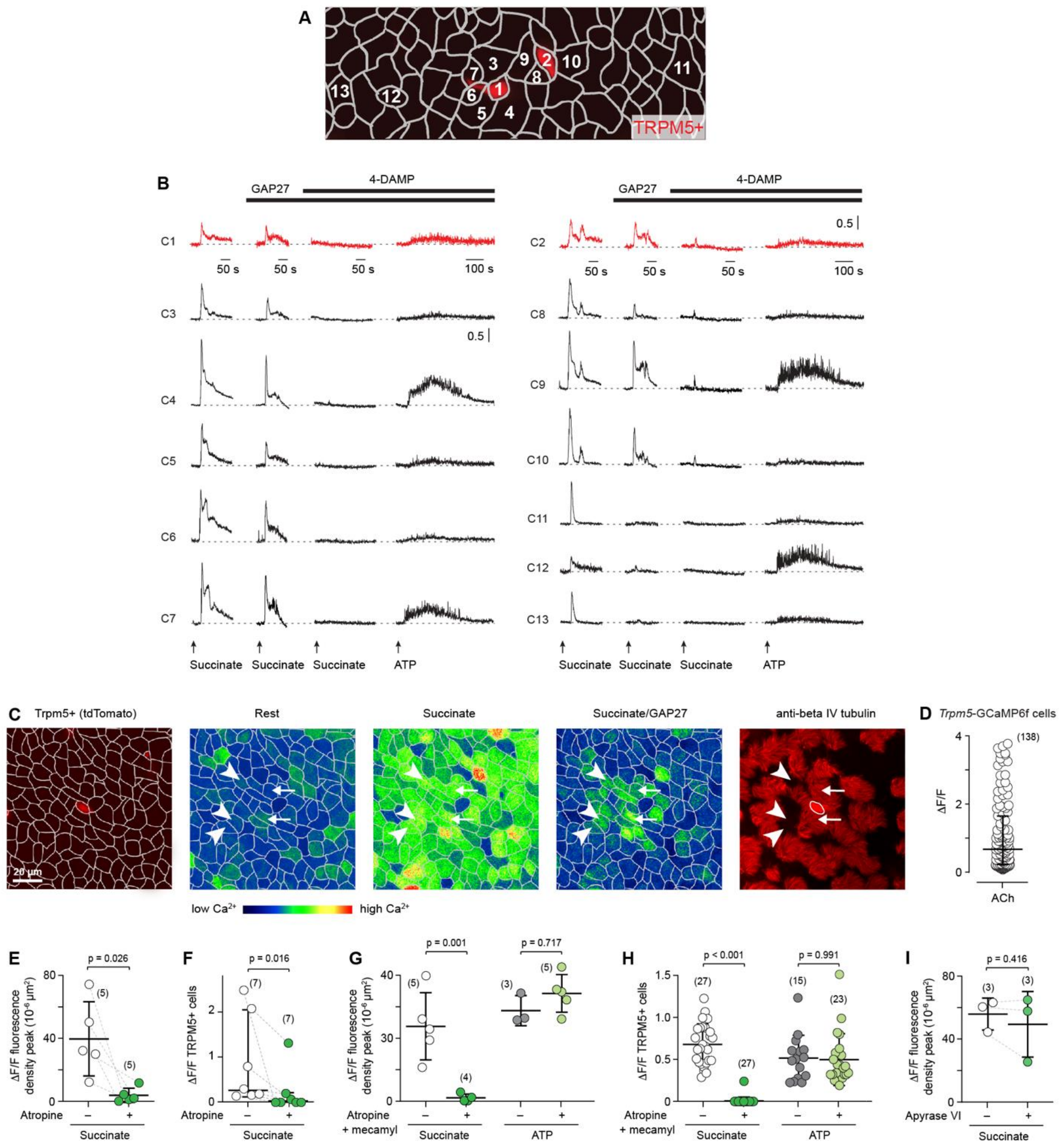
A
TRPM5+
B
GAP27
4-DAMP
C1
C2
C3
C4
C5
C6
C7
C8
C9
C10
C11
C12
C13
50 s
100 s
0.5
Succinate
ATP
C
Trpm5+ (tdTomato)
Rest
Succinate
Succinate/GAP27
anti-beta IV tubulin
20 µm
low Ca2+
high Ca2+
D
Trpm5-GCaMP6f cells
ΔF/F
ACh
E
p = 0.026
ΔF/F fluorescence density peak (10⁻⁶ µm²)
Atropine
Succinate
F
p = 0.016
ΔF/F TRPM5+ cells
G
p = 0.001
p = 0.717
Atropine + mecamyl
H
p < 0.001
p = 0.991
I
p = 0.416
Apyrase VI

Figure S6

Fig. S6. Validation of tracheal single-cell Ca^{2+} responses evoked by succinate or ATP before and after gap junction inhibition or muscarinic receptor blockade. (**A**) Confocal image indicating the location of $TRPM5^{+}$ cells (tdTomato, red) and their adjacent cells in the tracheal epithelium of a *Trpm5*-tdTomato mouse. Borders of the tracheal epithelial cells are indicated in light grey. This image is a higher magnification of the region delimited by the white box shown in Fig. 4B. Numbers indicate the spatial locations of the cells analyzed in B. (**B**) Time courses of original Ca^{2+} responses in individual cells (C1 to C13) evoked by succinate or ATP exposure under control conditions, after treatment with GAP27 (130 μM), and during additional treatment with 4-DAMP (1 μM). (**C**) Confocal images indicating the location of $TRPM5^{+}$ cells (tdTomato, red) and their adjacent cells, during Cal520 measurements at rest and at peak succinate (1 mM) response under control and GAP27 conditions, followed by posthoc immunostaining with anti-beta IV tubulin (red) in the tracheal epithelium of a *Trpm5*-tdTomato mouse. Cal520 images are mean peak Ca^{2+} waves, which represent the mean fluorescence intensities of 18 frames (512 x 512 pixels, $\Delta t = 0.74$ s, time frame = 13.32 s). Borders of the tracheal epithelial cells are indicated in light grey. GAP27-independent, succinate-evoked Ca^{2+} responses were observed in both ciliated (white arrow) and non-ciliated cells (white arrowheads). The $TRMP5^{+}$ cell identified by tdTomato (red) fluorescence can still be observed after the posthoc immunostaining (white oval circle in the anti-beta IV tubulin image). (**D**) Summary of Ca^{2+} peak values in individual Trpm5-GCaMP6f brush cells (138/163 cells; 84.7%) responding to acetylcholine (ACh, 100 μM). Median ± IQR. (**E**) Group data of peak ΔF/F density values of succinate-evoked Ca^{2+} waves before and after treatment with atropine (10 μM). Mean ± SD. Paired t-test. (**F**) Group data of succinate-evoked ΔF/F Ca^{2+} responses of individual $TRPM5^{+}$ cells before and after treatment with atropine (10 μM). Data, median ± IQR. Wilcoxon signed-rank test. (**G, H**) Group data showing peak ΔF/F density values of succinate-evoked Ca^{2+} waves (F) and of ΔF/F values of individual $TRPM5^{+}$ cells (G) before and after treatment with a mixture of atropine (10 μM) and mecamylamine (100 μM). ATP (20 μM) served as a control stimulus. ANOVA, posthoc: Tukey. (**I**) Group data of peak ΔF/F density values of succinate-evoked Ca^{2+} waves before and after treatment with apyrase VI (10 U/ml). Data, mean ± SD. Paired t-test. Succinate 1 mM; ATP 20 μM. Numbers in parentheses indicate independent experiments.

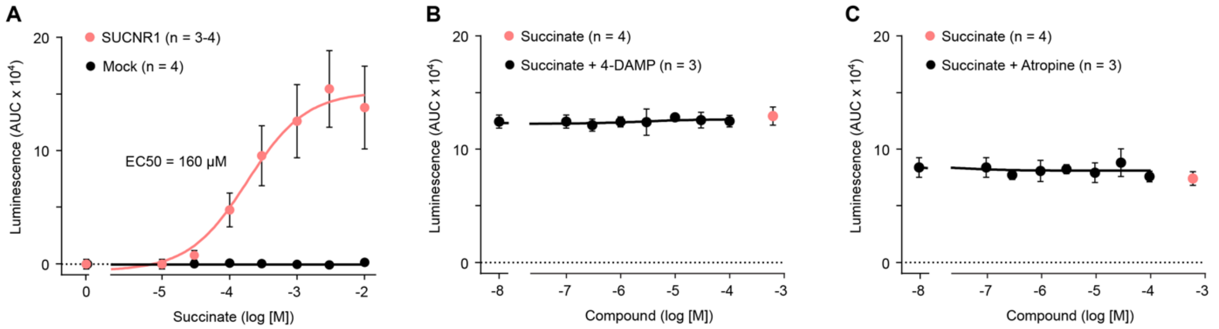

Fig. S7. Muscarinic inhibitors 4-DAMP and atropine do not affect succinate-induced SUCNR1 activity. (A) Changes in $[Ca^{2+}]_i$ depicted as luminescence of HEK293 cells transfected with plasmids coding for the human SUCNR1 or an empty vector (mock) upon stimulation with various concentrations of succinate. **(B-C)** Effect of the muscarinic antagonists 4-DAMP (B) and atropine (C) on SUCNR1 activity upon stimulation with succinate (650 µM, approx. EC_{80}), depicted as as luminescence of SUCNR1 transfected HEK293 cells. Data, mean ± SEM. Numbers in parentheses indicate measured wells for each condition. Data are obtained from at least two independent experiments.

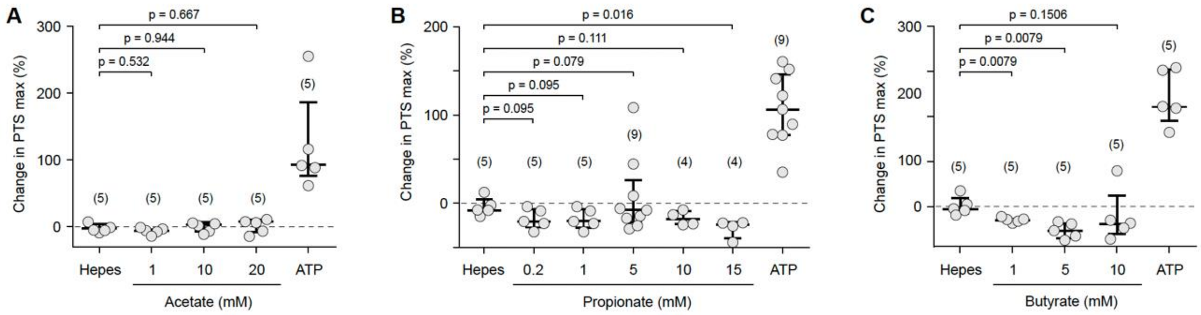

Fig. S8. Short chain fatty acids do not accelerate PTS. (**A to C**) Maximum changes in PTS in percent evoked by increasing concentrations of (A) acetate, (B) propionate, and (C) butyrate. Data, median ± IQR, number of tracheas is given in parentheses; Mann-Whitney-test.

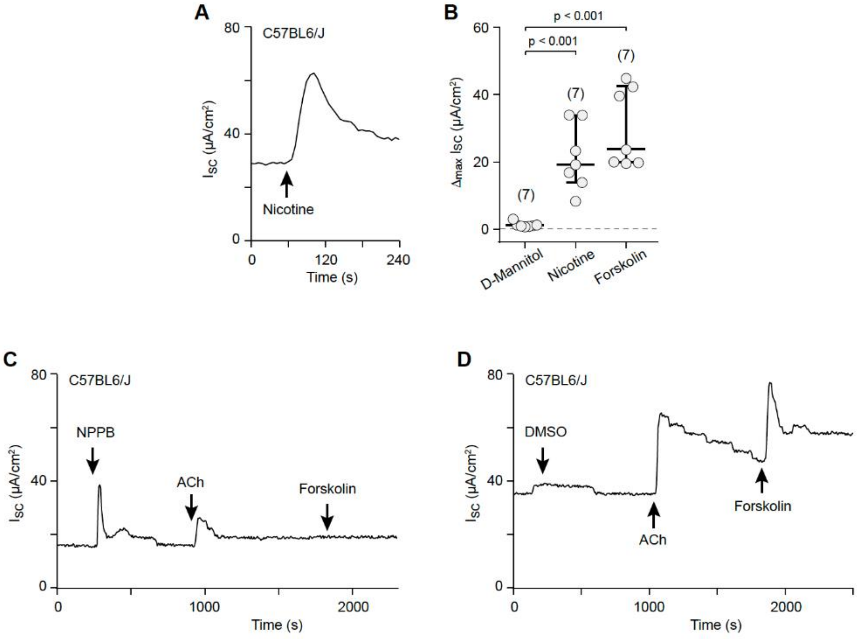

Fig. S9. Validation of the mouse tracheal Ussing chamber preparation. (**A**) Representative recording of transepithelial current (I_{SC}) in an opened mouse trachea mounted in an Ussing chamber and stimulated with nicotine (100 µM). (**B**) Maximum increases in transepithelial current (ΔI_{SC}) recorded in experiments as shown in A utilizing the nicotinic ACh receptor agonist nicotine (100 µM), the adenylate cyclase activator forskolin (10 µM), and D-mannitol (1 mM) as osmolarity control. Data, median ± IQR, Mann-Whitney-test, number of tracheas given in parentheses. (**C and D**) Representative recordings of experiments summarized in Fig. 7F showing the effect of (C) the Cl^- channel inhibitor NPPB (100 µM) and (D) its vehicle DMSO (500 µM) on I_{SC} and on currents induced by ACh (100 µM) and forskolin (10 µM).

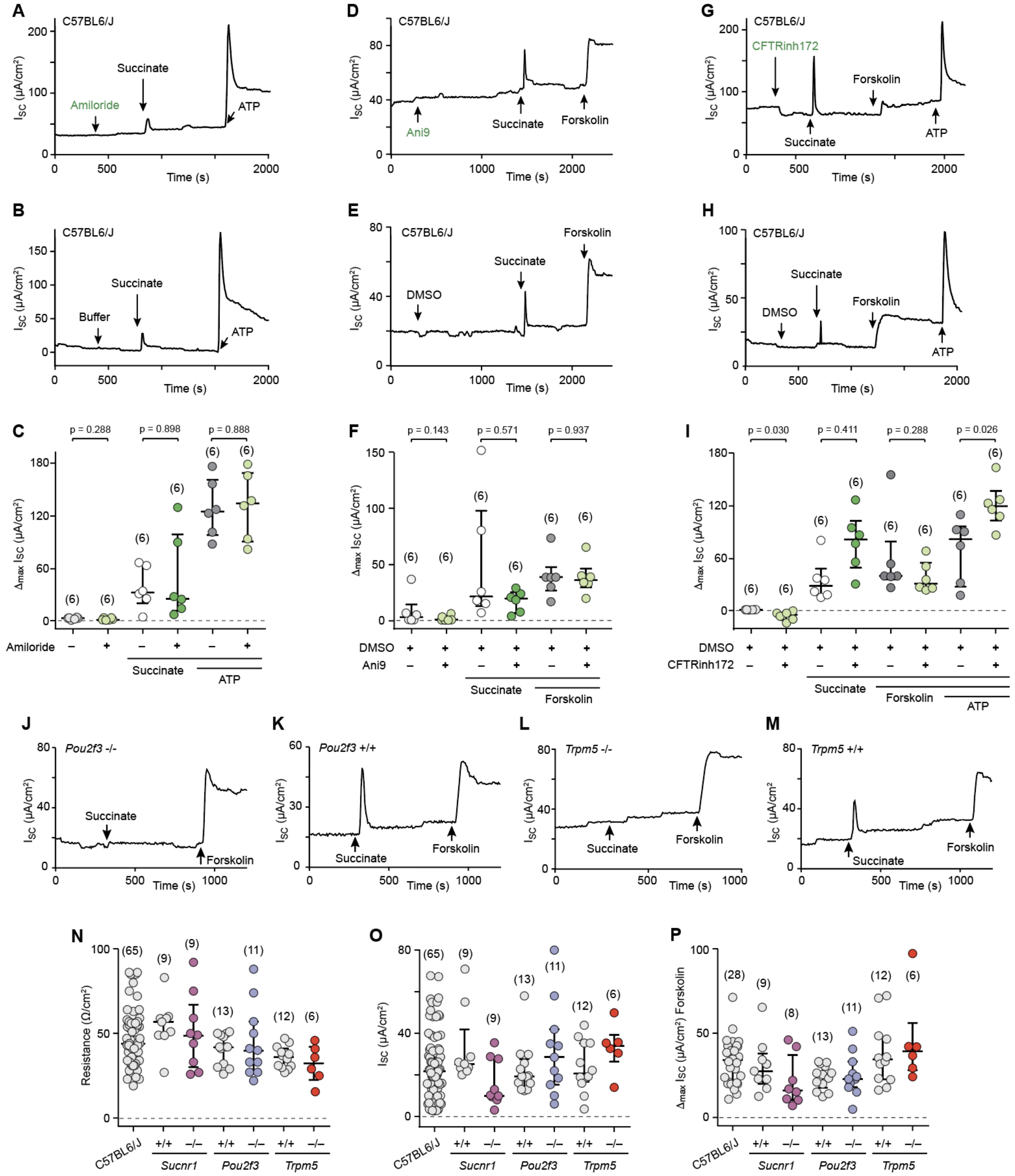

A
B
C
D
E
F
G
H
I
J
K
L
M
N
O
P
C57BL6/J
Succinate
Amiloride
ATP
Buffer
Ani9
Forskolin
DMSO
CFTRinh172
Time (s)
Pou2f3 -/-
Pou2f3 +/+
Trpm5 -/-
Trpm5 +/+
Sucnr1
Pou2f3
Trpm5
Resistance (Ω/cm²)
p = 0.288
p = 0.898
p = 0.888
p = 0.143
p = 0.571
p = 0.937
p = 0.030
p = 0.411
p = 0.288
p = 0.026

Figure S10

Fig. S10. A SUCNR1-brush cell pathway drives transepithelial currents independent of sodium channels or CFTR. (**A, B, D, E, G, and H**) Representative recordings of transepithelial current (I_{SC}) in an opened mouse trachea mounted in an Ussing chamber and exposed to succinate (1 mM), **(C, F, and I)** group data showing maximum increases in transepithelial current (ΔI_{SC}). ATP (100 µM, A to C and G to I) and the adenylate cyclase activator forskolin (10 µM, D to I) served as controls for vitality of the preparation. Vehicle controls were buffer for experiments with the sodium channel inhibitor amiloride (100 µM) (A to C) and DMSO (500 µM) for the experiments with the TMEM16A inhibitor Ani9 (10 µM, D to F) and with the CFTR inhibitor CFTRinh-172 (25 µM) (G to I). (**J-M**) Representative recordings of I_{SC} from experiments depicted as group data in Fig. 7M (J and K) and 7N (L and M), respectively. (**N to P**) Group data showing baseline tracheal epithelial resistance (N), baseline short-circuit current (O), and its increase in response to forskolin (10 µM) (P) in mouse strains used in this study. Representative recordings of experiments with tracheas from *Sucnr1*$^{-/-}$ and *Sucnr1*$^{+/+}$ mice are shown in Fig. 7J and K, respectively. Data, median ± IQR, number of tracheas in parentheses, Mann-Whitney-test in (C, F and I) and Kruskal-Wallis-test in (N to P).

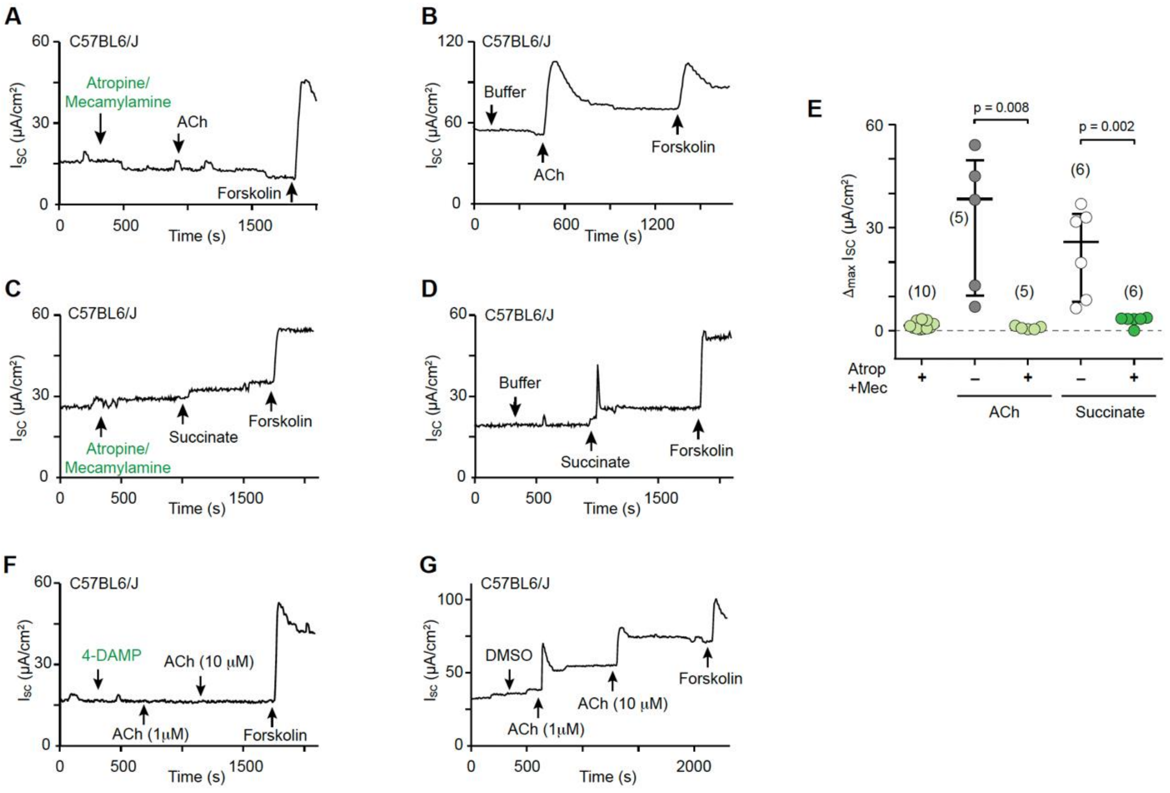

Fig. S11. Cholinergic receptor blockade inhibits both ACh- and succinate-induced Cl^- secretion. (**A to E**) Effect of combined nicotinic and muscarinic receptor blockade by mecamylamine (25 µM) and atropine (25 µM) (A and C) compared to their vehicle (buffer; B and D) on transepithelial currents induced by ACh (A and B) and succinate (1 mM) (C and D). (A to D) representative recordings from explanted tracheas, and (E) and group data showing maximum increases in ΔI_{SC}. (**F and G**) Representative recordings of experiments summarized in Fig. 8A showing the effect of (F) the muscarinic M3 receptor inhibitor 4-DAMP (1 µM) and (G) its vehicle DMSO (1 mM) on I_{SC} and on currents induced by ACh (100 µM). In all experiments, forskolin (10 µM) served as a brush cell-independent control stimulus. All inhibitors were applied both apically and basolaterally. Data, median ± IQR, number of tracheas in parentheses, Mann-Whitney-test.

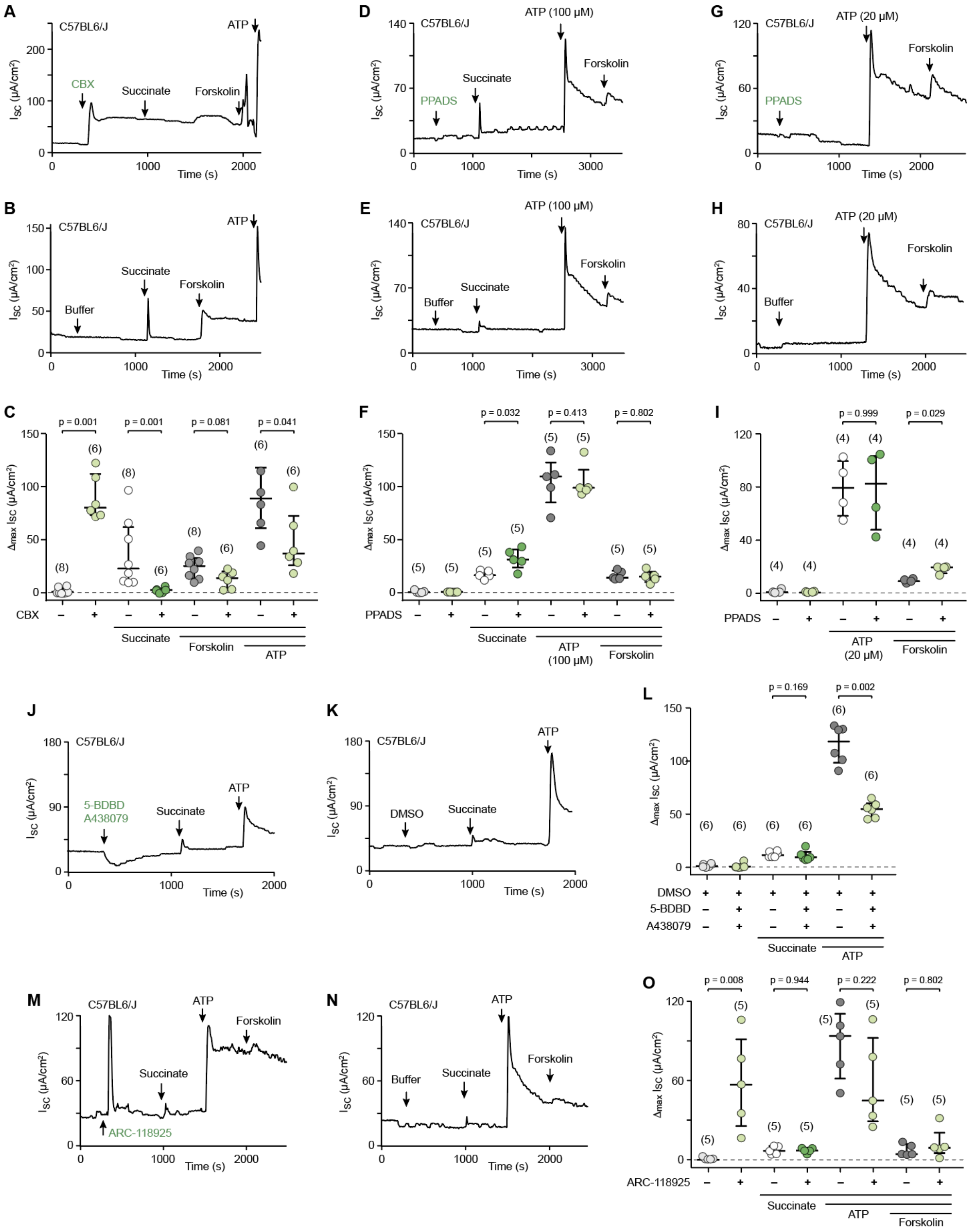
A
C57BL6/J
CBX
Succinate
Forskolin
ATP
B
Buffer
D
ATP (100 µM)
PPADS
G
ATP (20 µM)
E
H
C
F
I
J
5-BDBD
A438079
K
DMSO
L
M
ARC-118925
N
O
Time (s)
p = 0.001
p = 0.081
p = 0.041
p = 0.032
p = 0.413
p = 0.802
p = 0.999
p = 0.029
p = 0.169
p = 0.002
p = 0.008
p = 0.944
p = 0.222

Figure S12

Fig. S12. Succinate-induced Cl^- secretion is blocked by gap junction inhibition but not by P2 receptor inhibition. (**A, B, D, E, G, H, J, K, M and N**) Representative recordings of transepithelial current (I_{SC}) in an opened mouse trachea mounted in an Ussing chamber and exposed to succinate (1 mM), ATP (100 µM, except for G to I: 20 µM), and forskolin (10 µM) as indicated. (**C, F, I, L and O)**. Group data showing maximum increases in transepithelial current (ΔI_{SC}). Vehicle control for experiments with the gap junction blocker carbenoxolone (CBX, 100 µM) (A to C) and with the P2 receptor inhibitor PPADS (100 µM) (D to I) was buffer, that for experiments with P2X4 and P2X7 inhibitors 5-BDBD (10 µM) and A438079 (20 µM) (J to L) and with the P2Y2 inhibitor ARC-118925 (10 µM) (M to O) was DMSO (500 µM). All inhibitors were applied both apically and basolaterally. Data, median ± IQR, number of tracheas is given in parentheses; Mann-Whitney-test.

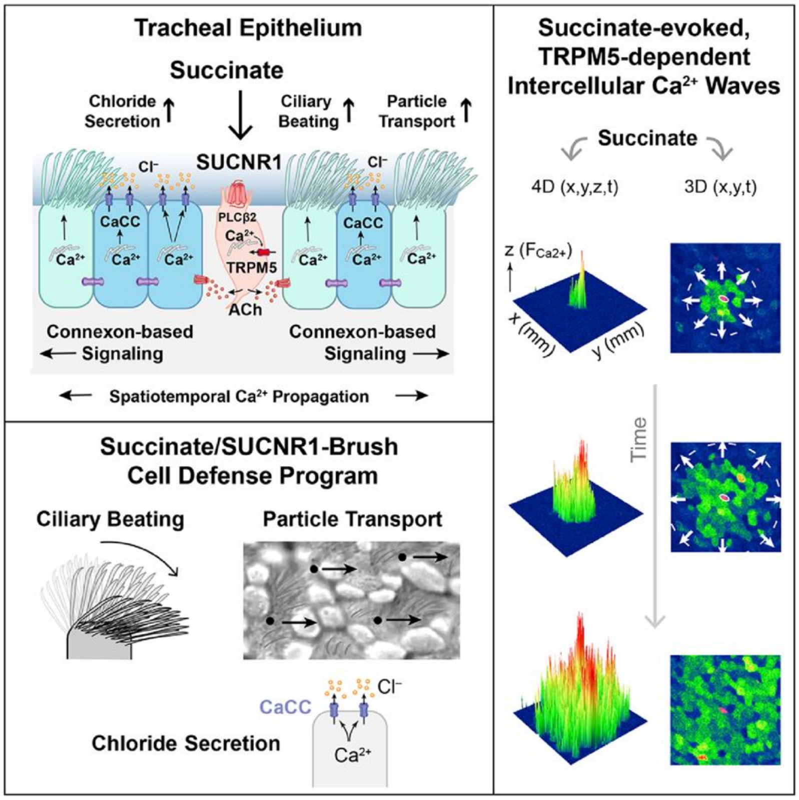

Fig. S13. A succinate/SUCNR1-brush cell defense program in the tracheal epithelium. This study proposes a model in which brush cells serve as a central hub after sensing luminal succinate to orchestrate a defense program in the trachea involving increased mucociliary clearance and Cl^- secretion driven by a Ca^{2+} wave propagating via sequential paracrine cholinergic and long-range gap junction signaling.

Table S1. Primer used for RT-PCR.

Target Gene (Accession Number)	Primer Sequence (5´-3´)	Product length
Sucnr1 (NM_032400.2)	fwd: *5´*- AGACAGAAGCCGACAGCAGAAT´<br>rev: 5´- GGTGTAGAGGTTGGTGTGAAGC´	332 bp
Trpm5 (NC_000073.7)	fwd: *5´-TATGGCTTGTGGCCTATGGT*-3´<br>rev: 5´-ACCAGCAGGAGAATGACCAG-3´	235 bp
GFP	fwd: 5´-AAGTTCATCTGCACCACCG-3´<br>rev: 5´-TCCTTGAAGAAGATGGTGCG-3´	187 bp
β-Actin (NC_000071.7)	fwd: 5´-CCATCATGAAGTGTGACGTTGA-3´,<br>rev: 5´-CATCGTACTCCTGCTTGCTGAT-3´	249 bp

Supplementary Videos

Video S1. Example of a succinate-induced intercellular Ca^{2+} wave in mouse tracheal epithelium. Succinate (1 mM) induced a long-range, intercellular Ca^{2+} wave that expanded in a circular pattern from a central initiating or trigger cell, the $TRPM5^{+}$ brush cell (red fluorescence), across the tracheal epithelium. The epithelium was loaded with the Ca^{2+} indicator dye Cal-520. The field of view is 123 µm × 123 µm (512 × 512 pixels). Each image is 1 frame acquired by scanning at 1.35 Hz. The speed of the movie was increased by 10x.

Video S2. Example of ATP-induced Ca^{2+} mobilization in mouse tracheal epithelium. The epithelium was loaded with the Ca^{2+} indicator dye Cal520. The field of view is 123 µm × 123 µm (512 × 512 pixels). Each image is 1 frame acquired by scanning at 1.35 Hz. The speed of the movie was increased by 10x. ATP, 20 µM. Same preparation as in Video S1.
